# Confinement-induced chirality in phase-separated achiral polymer solutions

**DOI:** 10.1126/sciadv.adv6734

**Published:** 2025-07-09

**Authors:** Baichuan Kou, Jin-Sheng Wu, Yingshan Ma, Yuhang Huang, Xiao He, Tianyi Wu, Zhi-Bo Yang, Paul van der Schoot, Ivan I. Smalyukh, Eugenia Kumacheva

**Affiliations:** ^1^ Department of Chemistry, University of Toronto, Toronto, ON M5S 3H6, Canada.; ^2^ Department of Physics and Chemical Physics Program, University of Colorado, Boulder, CO 80309, USA.; ^3^ Department of Chemical Engineering and Applied Chemistry, University of Toronto, Toronto, ON M5S 3E5, Canada.; ^4^ Department of Applied Physics and Science Education, Eindhoven University of Technology, P.O. Box 513, 5600 MB Eindhoven, Netherlands.; ^5^ Department of Electrical, Computer, and Energy Engineering, Materials Science and Engineering Program and Soft Materials Research Center, University of Colorado, Boulder, CO 80309, USA.; ^6^ Renewable and Sustainable Energy Institute, National Renewable Energy Laboratory and University of Colorado, Boulder, CO 80309, USA.; ^7^ International Institute for Sustainability with Knotted Chiral Meta Matter (WPI-SKCM^2^), Hiroshima University, Higashi-Hiroshima, Hiroshima 739-8526, Japan.; ^8^ Institute of Biomedical Engineering, University of Toronto, Toronto, ON M5S 3G9, Canada.

## Abstract

Self-organization of polymers in constrained geometries largely determines their applications in high-strength materials, photonics, and electronics. Chiral organization under confinement is well established for polymers with intrinsic molecular chirality; however, it has not been observed for achiral polymers. Here, we report the emergence of chirality in spatially confined solutions of achiral rigid-rod polymers. We show that kinetically arrested phase separation of polymer solutions confined to narrow capillaries resulted in alternating segments of isotropic and chiral nematic phases. The chiral structure of the nematic segments originated from the interplay between the constrained geometry, surface anchoring, orientational wetting, and elastic anisotropy of rigid-rod polymers. The catenoidal shape of the chiral structure recapitulated the morphology of biological chiral structures. These findings provide insight into the organization of soft matter under spatial confinement and offer a straightforward way to form chiral structures from achiral synthetic polymers.

## Teaser

Achiral rigid-rod polymers self-organize into chiral structures under confinement.

## INTRODUCTION

The organization of macromolecules in spatial confinement is one of the key areas in fundamental research and related polymer applications (*1*, *2*). Spatial confinement signifies the importance of polymer-surface interactions and imposes geometric constraints on polymer conformation and orientation, leading to distinct modes of self-assembly, phase separation, and crystallization. Self-assembly of block copolymers in small particles or thin films gives rise to nanometer-scale topological and chemical patterns with a high degree of order and complexity (*3*). Polymer crystallization in constrained geometries leads to distinct orientation, morphology, and crystallization kinetics of polymer melts and solutions (*4*). Surface tension–driven or buoyancy-driven instabilities in polymer films produce a great wealth of spatiotemporal topographic and compositional patterns (*5*, *6*).

Spatial confinement also enhances the organization of rigid-rod or semiflexible polymers in liquid crystalline phases (*7*–*9*). The orientational order arises from the molecular shape anisotropy (*10*–*12*) and is controlled by the constrained geometry and polymer surface anchoring (*8*, *9*, *12*). Furthermore, chirality transfer over several length scales has been elucidated for DNA origami filaments (*13*), protein fibrils (*14*), cellulose assemblies (*15*–*20*), and filamentous viruses (*21*), all formed by polymers with substantial rigidity and intrinsic molecular chirality. Despite intense interest in creating chiral structures from achiral objects (*22*–*28*), no strategies have been reported for achiral polymers. However, based on recent studies on disk-shaped surfactant micelle liquid crystals (*29*) and chromonic liquid crystals formed by rodlike aggregates of amphiphilic aromatic compounds (*30*–*37*), both exhibiting large elastic anisotropy (*38*), it can be hypothesized that, to minimize the elastic free energy of the system, confined rigid-rod polymers with large elastic anisotropy (*39*–*41*) can undergo spontaneous twist deformation and organize into chiral structures. This mechanism was theoretically predicted for rigid-rod polymers (*42*, *43*) but has not been experimentally explored.

Here, we focused on a synthetic water-soluble achiral rodlike polyelectrolyte, poly(2,2′-disulfonyl-4,4′-benzidine terephthalamide) (PBDT), which, in macroscopic (unconstrained) solutions, undergoes phase separation into isotropic phase and nematic liquid crystalline phase (*44*–*46*). We show that when confined to narrow capillaries, PBDT solutions exhibit kinetically arrested phase separation into alternating isotropic and nematic liquid crystalline segments with scale-invariant segment lengths. While PBDT does not have molecular chirality, the nematic segments acquired chiral structures that recapitulate the catenoidal twisted morphology of biological chiral structures formed by collagen fibrils (*47*, *48*), nucleosome core particles (*49*, *50*), and filamentous viruses (*51*). Our experimental results, numerical simulations, and scaling analysis demonstrated that the spontaneous formation of chiral nematic segments stemmed from three intrinsic properties of rigid-rod polymers: (i) curvature-sensitive surface anchoring, (ii) orientational wetting against a solid surface, and (iii) large elastic anisotropy that favors twist deformation. Our findings provide insight into the origin of structural chirality from nature-derived polymers and pave the way for chirality induction in man-made polymer materials.

## RESULTS

### Phase separation under confinement

**Fig. 1.**
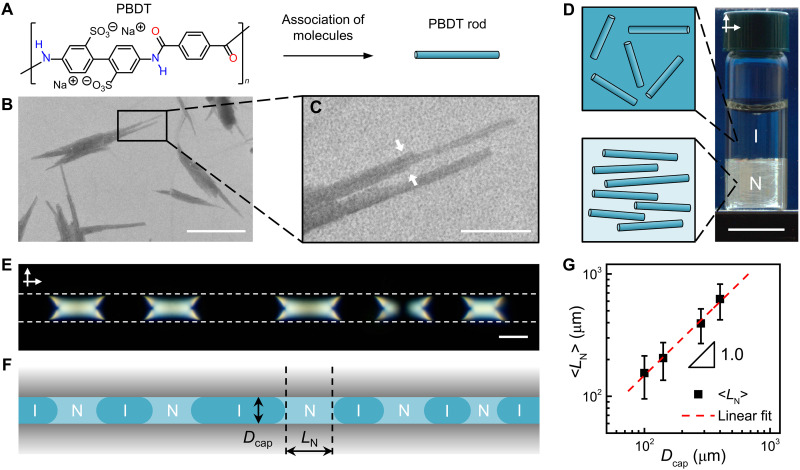
NI phase separation of PBDT solution. (**A**) Chemical structure of PBDT. (**B**) TEM image of PBDT rods deposited from the polymer solution at *C*_P_ = 0.01 wt % and negatively stained with uranyl acetate. Scale bar, 200 nm. (**C**) Enlarged image of the area marked in (B) with the black rectangle. Scale bar, 50 nm. (**D**) Photograph of the 1.7 wt % phase-separated PBDT solution viewed between crossed polarizers. The corresponding schematic shows the rod orientation in the nematic (N) and the isotropic (I) phases. Scale bar, 1 cm. (**E**) POM image of the PBDT solution as in (D) confined to a 100-μm-diameter capillary. The dashed white lines outline the glass surface. The arrows show the polarization directions of crossed polarizer and analyzer. The capillary is aligned parallel to the polarizer. Scale bar, 100 μm. (**F**) Schematic of the isotropic and nematic segments as in (E) with nematic segment length *L*_N_ in the capillary with diameter *D*_cap_. (**G**) Variation in the average nematic segment length <*L*_N_>, plotted as a function of *D*_cap_. The data were obtained from about 500 to 1000 nematic segments for each capillary diameter. The error bars represent the standard deviations of *L*_N_. The dashed red line shows the linear fitting of the results.

Synthesis of PBDT with a chemical structure shown in Fig. 1A was carried out by interfacial polycondensation reaction (*44*). The details of PBDT synthesis, fractionation, and characterizations are provided in Materials and Methods. In dilute aqueous solutions without added salt, PBDT exists as semirigid molecules that, above a crossover polymer concentration, undergo side-by-side assembly to form negatively charged rigid rods with persistence length exceeding 1 μm (*45*, *46*). A representative transmission electron microscopy (TEM) image (Fig. 1B) shows bundles of PBDT rods formed in the presence of a staining agent, in agreement with the reported counter ion–induced bundling of rodlike polyelectrolyte (*52*). The enlarged image (Fig. 1C) shows a bundle formed by three PBDT rods (pointed with white arrows). The rod diameter, measured from TEM images, was found to be 1.1 ± 0.2 nm (section S1).

At PBDT concentration (*C*_P_) of 1.3 wt % < *C*_P_ < 2.5 wt %, the polymer solution phase separated into an isotropic (top) phase and a nematic liquid crystalline (bottom) phase (Fig. 1D and fig. S2), with the volume fraction of the nematic phase (Φ_N_) increasing with *C*_P_ (fig. S3). The broad range of *C*_P_ in the biphasic region was attributed to the polydispersity in the length of the PBDT rods. This is because, upon phase separation, longer PBDT rods partitioned into the nematic phase, while shorter rods remained in the isotropic phase (*10*, *53*). On the basis of the Onsager theory with electrostatic interactions taken into account, we estimated the length of the PBDT rods to be in the range of 75 to 94 nm (section S1). The achiral nematic nature of the liquid crystalline phase was confirmed by its characteristic Schlieren texture observed using polarized optical microscopy (POM) (fig. S4).

A PBDT solution with *C*_P_ = 1.7 wt % was introduced into cylinder-shaped glass capillaries with an inner diameter (*D*_cap_) in the range of 100 to 400 μm to achieve a varying degree of confinement of the polymer solution (fig. S5). Following a 7-day equilibration (fig. S6), the state of the solution was examined using POM. In comparison with macroscopic phase separation (Fig. 1D), spatial confinement led to the formation of alternating isotropic (dark) and nematic liquid crystalline (bright) segments (Fig. 1E). As illustrated in the schematic in Fig. 1F, the nematic segments exhibited strong wetting at the nematic-solid (NS) interface and formed concave-shaped menisci at the nematic-isotropic (NI) interface.

The dimensions and shapes of the nematic segments did not change for at least 4 months, indicating that the phase separation of the polymer solution was kinetically arrested (*54*, *55*). While in the unconstrained PBDT solution, gravity governed the sedimentation of the nematic phase, the effect of gravity in the capillaries was negligible (fig. S7), compared to the interfacial tension of the NI interface and the friction at the liquid-solid interface. Nematic segments with different lengths (*L*_N_) formed menisci with similar curvatures at the interface with the isotropic phase, which prevented segment coarsening driven by Laplace pressure (*54*).

For capillaries with *D*_cap_ of 100 to 400 μm, the variation in the average nematic segment length, <*L*_N_>, versus *D*_cap_ followed a scaling relationship of <*L*_N_> ~ 1.54*D*_cap_, indicating that the nematic segment length increased linearly with the capillary diameter, while the ratio <*L*_N_>/*D*_cap_ remained constant (Fig. 1G and figs. S5 and S9A). The average isotropic segment length <*L*_I_> also showed a linear scaling relationship of <*L*_I_> ~ 2.23*D*_cap_ with *D*_cap_ (figs. S9B and S10). Thus, the relative segment lengths (<*L*_N_>/*D*_cap_ and <*L*_I_>/*D*_cap_) remained scale-invariant under confinement. The volume fractions of the confined nematic and isotropic segments, calculated from the segment lengths, matched those determined from macroscopically phase-separated samples (section S2 and figs. S11 to S13). We note that the linear scaling relationship found in our study is different from the results of a previous study on capillary-confined liquid-liquid phase separation, where a scaling exponent of 1.3 was found for a larger range of *D*_cap_ (*56*).

### Chirality of nematic segments

**Fig. 2.**
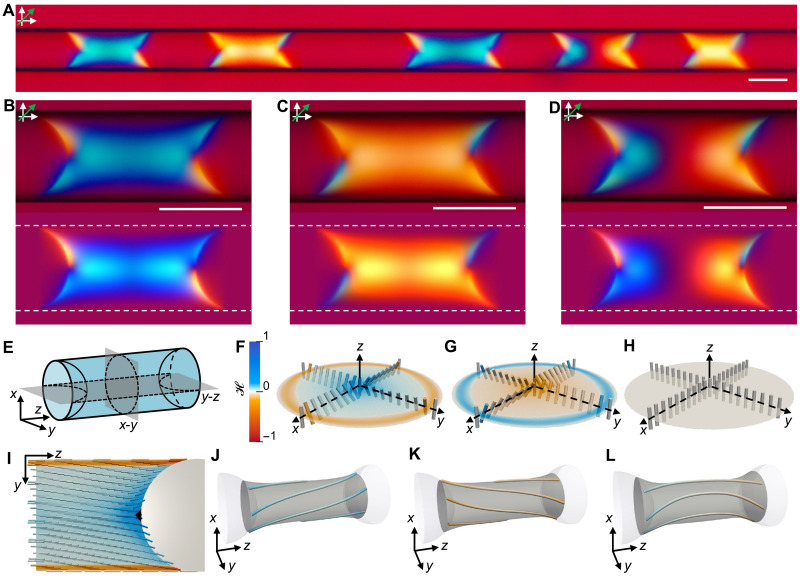
Chirality of nematic segments. (**A**) POM image of the nematic segments as in Fig. 1E viewed under crossed polarizer and analyzer with inserted full-wave plate (λ = 530 nm). *D*_cap_ = 100 μm. *C*_P_ = 1.7 wt %. The green arrow indicates the slow axis of the full-wave plate. (**B** to **D**) Experimental (top) and simulated (bottom) POM images of (B) a right-handed segment, (C) a left-handed segment, and (D) a segment with opposite twists at the two ends. The dashed white lines outline the glass surface. (**E**) Schematic of the segment geometry (*L*_N_ = 1.54*D*_cap_; *D*_cap_ = 100 μm) used in simulations. The middle transverse cross section (*x*-*y*) and the longitudinal cross section (*y*-*z*) are marked by dashed black curves. The middle transverse cross section is located at the midplane of the nematic segment. (**F** to **H**) Simulated director field of the middle transverse cross section showing (F) a right-handed twist, (G) a left-handed twist, and (H) a uniform axial director field. The cross sections are color coded based on the handedness parameter H . (**I**) Simulated longitudinal cross section of the right-handed chiral nematic segment. The defect is marked by the black region at the center of the NI interface. (**J** to **L**) Three-dimensional streamlines of the simulated (J) right-handed segment, (K) left-handed segment, and (L) segment with opposite twists at the two ends. The streamlines lie on the gray catenoidal surface and are color coded based on the handedness parameter H . The uniaxial director lines at the central axis of the capillary and at the glass surface are not shown in (J) to (L). Scale bars, 100 μm.

To visualize the orientation of the PBDT rods in the nematic segments, we inserted a full-wave plate (λ = 530 nm) between the sample and the analyzer, which revealed distinctive interference colors of the segments (Fig. 2A). The twisted orientation of the rods in the main body of these segments was evidenced by the blue or yellow colors (Fig. 2, B to D, top). The different interference colors indicated that the major axis of the transmitted light was rotated in different directions, being governed by the twist handedness (section S3 and fig. S14). Because PBDT solutions have positive birefringence (Δ*n* = 0.004 for the nematic phase of the 1.7 wt % PBDT solution; Materials and Methods), we inferred that the blue and yellow colors corresponded to right- and left-handed twists, respectively. The fractions of the right- and left-handed segments were approximately equal (fig. S15), with 5% of the segments showing opposite twists at the two ends (Fig. 2D). The middle region of these segments appeared dark when the capillary was parallel to the polarizer but turned bright when the capillary was rotated by 45° (fig. S11), implying that the PBDT rods were locally orientated parallel to the capillary axis, that is, they were untwisted. At the interface with the isotropic phase, both blue and yellow colors were observed for the meniscus tips because of the preferred planar alignment of the PBDT rods at the NI interface, in agreement with the results of experimental (*45*) and theoretical studies (*57*).

Numerical simulations were performed to elucidate the chiral structure of the nematic segments. We applied a planar anchoring condition to both the NI interface and the NS interface and ensured that the polar anchoring strength of the rigid glass surface was higher than that of the fluid NI interface, which was observed for liquid crystalline dispersions of carbon nanotubes (*58*). Azimuthal anchoring at the glass surface was not enforced but emerged spontaneously in our simulations (Materials and Methods). Theoretical studies suggested that, for liquid crystals of rodlike polyelectrolytes, the splay (*K*_11_), twist (*K*_22_), and bend (*K*_33_) elastic constants, which describe the elastic free energy costs of the corresponding director field deformations, exhibit the relation *K*_33_ >> *K*_11_ = 3*K*_22_ (*41*). Experiments on rodlike particles, such as tobacco mosaic virus, yielded elastic constants in agreement with the theory (*40*). However, *K*_33_ ≈ *K*_11_ was found for carbon nanotubes (*59*), assuming that the saddle-splay elastic constant *K*_24_ is much smaller than *K*_11_ or *K*_22_ (Materials and Methods). For polymer liquid crystals, such as poly-γ-benzyl-l-glutamate, both *K*_11_ and *K*_33_ are an order of magnitude larger than *K*_22_ (*40*). Such elastic anisotropy induced mirror symmetry breaking in chromonic liquid crystals (*31*–*33*). Thus, we assumed that for the PBDT rods, *K*_11_ = *K*_33_ = 10 pN and *K*_22_ = 0.6 pN, that is, *K*_11_/*K*_22_ = *K*_33_/*K*_22_ = 16.7 (Materials and Methods).

Three equilibrium chiral structures were found by minimizing the total Landau–de Gennes free energy of the nematic segment with the geometry shown in Fig. 2E. Figure 2 (F to H) illustrates the middle transverse cross sections of the simulated chiral segments. The director at the central axis of the capillary was parallel to the capillary long axis; however, the configurations in Fig. 2 (F and G) show a right-handed twist and a left-handed twist, respectively, that started from the central axis of the capillary. The director field close to the central axis is illustrated in fig. S16A. The twist handedness was reversed as the director became parallel to the capillary long axis again at the glass surface, even though no azimuthal anchoring was involved in simulations. For the segment with opposite twists at the two ends (Fig. 2D), the director field adopted a uniaxial alignment (helical perversion) in the middle of the segment, which separated domains of opposite twists (Fig. 2H). The longitudinal cross section (Fig. 2I) shows that the director was also tilted at the concave-shaped meniscus, forming a point defect (boojum) at the center of the NI interface. The enlarged images of the director field close to the defect are shown in figs. S16B and S17. The cross sections were color coded using a handedness parameter, H , to show the spatial variation of the handedness and the magnitude of local twist deformation, with blue (H  > 0) and red (H  < 0) colors representing right and left handedness, respectively (Materials and Methods). The gradient in the blue color in Fig. 2I indicated a substantial twist deformation near the defect, which was the effect that was reported for nematic droplets and colloids (*31*, *32*). The appearance of the simulated POM images of the nematic segments (Fig. 2, B to D, bottom), based on the director field, was in agreement with experimental observations (Fig. 2, B to D, top).

The three-dimensional structures of the chiral segments were illustrated by drawing the streamlines of the simulated director field (Fig. 2, J to L). These lines were not only twisted but also curved, thus forming a catenoidal twist pattern. Similar catenoidal shapes have been observed for condensed assemblies of collagen fibrils (*47*, *48*), nucleosome core particles (*49*, *50*), and filamentous viruses (*51*), in which chiral rodlike objects were packed into doubly twisted bundles. In contrast, PBDT rods are achiral, yet they still form chiral structures, thus indicating a chirality induction mechanism that is ubiquitous to rigid rods. For the nematic segments formed by PBDT rods, the defect on the NI interface enforces substantial splay deformation in the surrounding director field. Because of the large elastic anisotropy that is intrinsic to rigid-rod polymers (*39*–*41*), the energetically more favorable twist deformation spontaneously arises to partially replace the splay deformation, thus reducing the total elastic free energy of the director field and leading to the structural chirality of nematic segments.

**Fig. 3.**
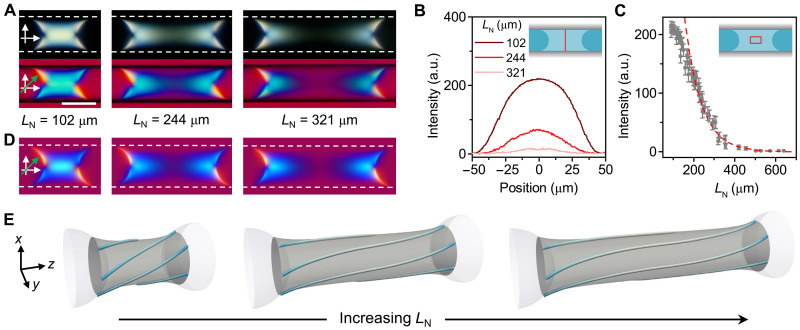
Propagation of chirality from the NI interface. (**A**) POM images of right-handed nematic segments with different lengths, taken between crossed polarizers (top) and with the full-wave plate (bottom). The dashed white lines outline the glass surface. *D*_cap_ = 100 μm. *C*_P_ = 1.7 wt %. (**B**) Light intensity profiles of the transverse cross sections at the midplane (marked by the vertical red line in the inset) of the nematic segments, measured in the POM images in the top panels in (A). a.u., arbitrary unit. (**C**) Variation in the light intensity measured in the central segment region plotted as a function of the segment length. The dashed red curve is the exponential decay fitting of the data with *L*_N_ in the range of 200 to 640 μm. The error bars are the standard deviations of the intensity measured in a rectangular region with dimensions (height × width) of 33 μm × *L*_N_/3 (marked by the red rectangle in the inset). (**D**) Simulated POM images of three nematic segments of different lengths taken with the full-wave plate. The dashed white lines outline the glass surface. (**E**) Streamlines on the catenoidal surfaces of the simulated segments in (D). The streamlines are color coded based on the handedness parameter H . Scale bar, 100 μm.

Figure 3 (A and B) shows the POM images of three nematic segments with varying *L*_N_ for which the defect was always present at the center of the NI interface. The light intensity of the middle transverse cross section of the nematic segment (Fig. 3B, inset) gradually decreased with increasing *L*_N_, which indicated gradual untwisting of the segments with increasing distance from the NI interface. This effect was prominent when *L*_N_ increased from 100 to 300 μm (Fig. 3C), and for the segments with *L*_N_ > 450 μm, the director field in the middle of the segments was completely untwisted. The observation of chirality only in the vicinity of the NI interface confirms that the structural chirality originated from the twist deformation near the defect.

The untwisting effect was reproduced in the simulated POM images (Fig. 3D) and their director streamlines (Fig. 3E). With increasing *L*_N_, the streamlines tended to align parallel to the capillary long axis in the middle of the segments, but remained twisted near the NI interface, thus resulting in a stretched catenoid surface (Fig. 3E). The preferred uniaxial alignment of PBDT rods in the middle of long nematic segments, that is, being far away from the NI interface, was also observed for the confined fully nematic solution at *C*_P_ = 2.5 wt % (fig. S18A).

### Orientational wetting

**Fig. 4.**
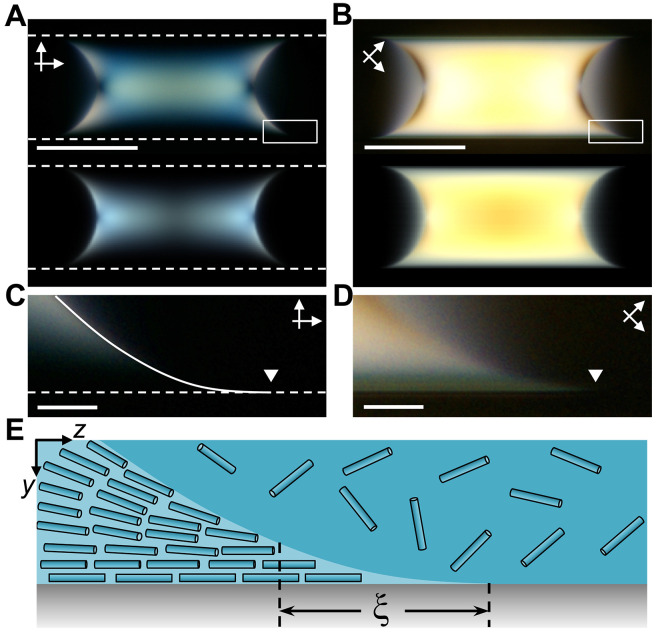
Orientational wetting at the meniscus tip of nematic segments. (**A** and **B**) Experimental (top) and simulated (bottom) POM images of the nematic segment as in Fig. 2B, acquired without the wave plate, with the capillary (A) aligned parallel to the polarizer and (B) rotated by 45°. The dashed white lines in (A) outline the glass surface. Scale bars, 100 μm. (**C** and **D**) Enlarged images of the meniscus tip marked with the white rectangles in (A) and (B), respectively. The location of the contact line is marked with white triangles. The dashed white line and solid white line in (C) show the glass surface and the NI interface, respectively. Scale bars, 10 μm. (**E**) Schematic of the longitudinal cross section of the meniscus tip of the nematic segment. ξ, the characteristic length scale.

Figure 4 (A and B) shows the experimental (top) and simulated (bottom) POM images of a nematic segment, acquired without the full-wave plate, with the capillary aligned parallel to the polarizer and rotated by 45°, respectively. The enlarged images of the meniscus tip, marked with the white rectangles in Fig. 4 (A and B), are shown in Fig. 4 (C and D), respectively, with the contact line marked with white triangles. The shape of the meniscus tip in Fig. 4D indicated complete wetting of the glass surface by the nematic phase. The dark appearance of the meniscus tip in Fig. 4C suggested that, in the nematic phase close to the nematic-isotropic-solid three-phase contact line, the PBDT rods were aligned parallel to the glass surface, in agreement with the simulation results (Fig. 2I). By reducing the concentration of the capillary-confined PBDT solution to 1.4 wt %, we obtained a thin nematic wetting film on the inner surface of the capillary with a uniaxial director field (fig. S19). On the basis of these observations, we propose that the PBDT rods exhibit strong orientational wetting on the glass surface, with a parallel rod alignment along the capillary long axis.

The orientational wetting of PBDT rods is expected, because it is a purely entropic effect driven by excluded volume interactions (*60*). To examine the role of hydrophobic and hydrophilic surface interactions, we confined PBDT solutions in a hydrophobically modified capillary with a water wetting angle of ~90° (section S4 and fig. S20). We found that the nematic phase was still able to wet the hydrophobic glass surface, although the wetting angle was greater than that on the untreated glass surface, and upon equilibration, chiral structures were also observed near the NI interface (fig. S21). Thus, we conclude that the wetting behaviors of PBDT rods and the formation of chiral structures are general effects for rigid-rod polymers, which are not critically affected by surface chemistries.

In the meniscus tip, the uniform alignment of PBDT rods parallel to the glass surface implied a stronger polar anchoring strength of the NS interface than that of the NI interface, which was observed for carbon nanotubes (*58*) and adopted in our simulations. The uniform alignment was only observed within a certain distance from the contact line; beyond this distance, the rods tended to align parallel to the NI interface (Fig. 4E). This characteristic length scale, ξ, of the region with uniform director field resulted from the competition between the elasticity of the director field and the surface anchoring at the NI interface (*61*–*63*). The uniform director field induced a small increase in the surface anchoring energy but avoided the energetically costly splay deformation and the formation of surface disclination at the contact line (*64*), thus reducing the total free energy. On the basis of a simple scaling theory (section S5), we correlated ξ with the splay elastic constant (*K*_11_), the anchoring strength of the NI interface (*W*_NI_), and the capillary diameter (*D*_cap_) and found the relation ξ ~ (*K*_11_*D*_cap_/*W*_NI_)^½^, which demonstrated the competition between elastic energy and surface anchoring energy, with *D*_cap_ representing the effect of geometric confinement. Moreover, the relative length of the uniformly aligned region, ξ/*D*_cap_ ~ (*K*_11_/*W*_NI_*D*_cap_)^½^, increased with decreasing *D*_cap_, which implied that, under stronger confinement effect, the elastic energy became the dominant factor in determining the director configuration. By measuring ξ in the capillaries with a diameter of 100 to 400 μm, we found a reasonable agreement between experimental results and the scaling predictions (section S5 and figs. S23 and S24).

### Dynamics of the formation of chiral nematic segments

**Fig. 5.**
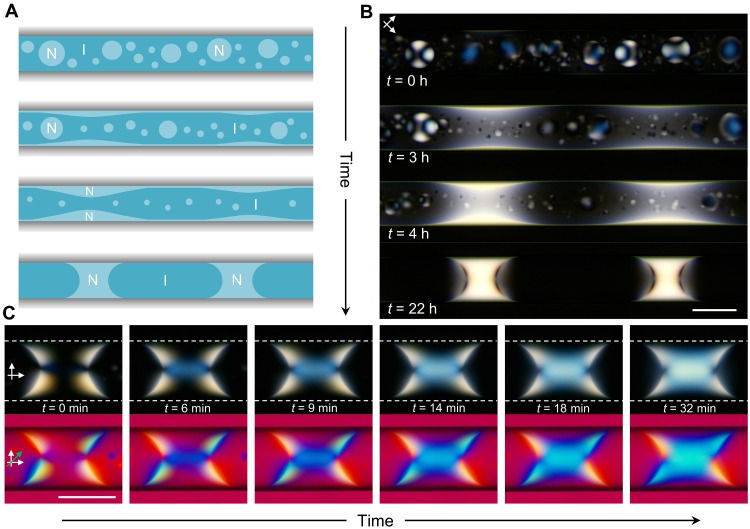
Decoupling of phase separation and emergence of chirality. (**A**) Schematic of the formation of nematic segments. Top to bottom: Nucleation of tactoids, formation of the nematic film, fluctuations in thickness of the nematic film, and formation of the nematic segments. (**B**) Top to bottom: Corresponding time-lapse POM images acquired during the course of the formation of nematic segments. (**C**) Evolution of chirality in a right-handed nematic segment. The top and the bottom rows show the time-lapse POM images acquired without and with the full-wave plate, respectively. The dashed white lines outline the glass surface. *D*_cap_ = 100 μm. *C*_P_ = 1.6 wt %. Scale bars, 100 μm.

The dynamic interplay between phase separation of the polymer solution and orientational wetting led to a distinct mechanism of the evolution of chiral nematic segments (Fig. 5, A and B, and movie S1). Small bipolar tactoids formed shortly after introducing the PBDT solution into the capillary. Orientational wetting of the glass surface by the nematic phase led to the formation of a thin nematic wetting film at the glass surface. Within 3 hours, the merging of tactoids with this film resulted in notable fluctuations in film thickness. The protruded film regions grew in thickness due to their further coalescence with tactoids and the capillary instability of the NI interface (*64*, *65*) and, ultimately, transformed into nematic segments. After ~24 hours, all tactoids disappeared, leaving mostly nematic segments spanning across the entire capillary (fig. S6).

For short nematic segments, phase separation and local mirror symmetry breaking were decoupled. Figure 5C illustrates the evolution of chirality in a nematic segment. Immediately after its formation, the segment did not exhibit discernible chiral features. The bright regions in the segment were characteristic of the preferred planar anchoring of the PBDT rods at the NI interface with the defect. Within 6 min, a blue interference color emerged from the defects, which was subsequently extended to form a cylindrical blue region. The blue-colored region gradually expanded toward the glass surface, and after ~30 min, a right-handed equilibrium chiral structure was formed. The fact that untwisted nematic segments formed first and later underwent a twisting transition suggested that either the thermodynamic driving force for this transition was weak or the reorientation kinetics for the PBDT rods were inherently slow, because of the high rotational viscosity of polymer liquid crystals (*40*). The evolution of chirality in nematic segments is in line with our prediction that to reduce the elastic energy, twist deformation would first occur near the surface point defect, where the energetically costly splay deformation can be partially relaxed by twisting the director field. We note that opposite twists, as shown in Fig. 2D, were not observed for short segments formed by the instability of thin nematic wetting film (fig. S11). The director relaxation process that leads to the domain wall-like structure in Fig. 2D remains a subject for further study.

## DISCUSSION

In this work, we have demonstrated the spontaneous mirror symmetry breaking of a rigid-rod polymer, namely, PBDT, confined to narrow capillaries. We found that the capillary-confined NI phase separation is crucial to the induction of chirality as it provides curved NI interface on which the point defect induces spontaneous twist deformation. The kinetically arrested phase separation leads to the formation of short nematic segments for which the length scales proportionally with the capillary diameter. Such phase separation constrains the twist deformation between the NI interfaces in proximity to each other, thus leading to a catenoidal twist pattern. We further showed that the orientational wetting properties of PBDT rods not only affect the equilibrium director field configuration but also disentangle the kinetics of phase separation and director relaxation that are driven by the shape anisotropy and elastic anisotropy of the rigid-rod polymer, respectively. It should be noted that, while the phase separation process is kinetically arrested, the chiral structures are formed under conditions of thermodynamic equilibrium. This effect distinguishes our work from recent reports on chiral structures obtained by meniscus-guided deposition (*24*, *27*, *28*, *66*) or under pressure-driven flow (*37*), where non-equilibrium factors such as solvent evaporation and shear forces may be at play.

In addition, we found that PBDT rods on the curved glass surface of a cylindrical capillary exhibit uniaxial planar anchoring along its long axis. This is distinct from the results obtained for chromonic liquid crystals, which prefer a surface director orientation along the circumference of the capillary (*34*, *35*, *67*). On one hand, we argue that the anisotropic excluded volume interactions between rigid rods and curved glass surface are expected to favor a rod alignment along the capillary long axis. On the basis of a simple scaling theory, we found that circumferential anchoring induces a small but nonnegligible increase in the interfacial tension of the NS interface (section S6). This rigid-rod model is applicable to PBDT rods because the average rod length (~100 nm) is much smaller than the persistence length (~1 μm). On the other hand, we note that on a curved glass surface, any director orientation that is not parallel to the capillary long axis inevitably induces a bend deformation in the director field, which increases the elastic free energy. Considering the large *K*_33_ of PBDT rods that is associated with the high bending rigidity, the uniaxial surface anchoring is also favored to avoid the bend deformation. The coupling between the director field and the surface curvature has been theoretically examined for cylindrically shaped thin nematic shells (*68*). Our observation of the uniform director field in both the meniscus tip of the nematic segments and the thin nematic wetting film provides experimental evidences that the nematic elasticity can align the director field at a curved surface.

The difference in surface anchoring gives rise to distinct mechanisms of spontaneous mirror symmetry breaking. For capillary-confined chromonic liquid crystals, twist-escaped configuration arises because of their large saddle-splay elastic constant *K*_24_ that favors circumferential anchoring (*34*, *35*, *67*). For PBDT rods, the chirality originates from the twist deformation near the defect on the NI interface, which is enabled by the small twist elastic constant *K*_22_ that is common for lyotropic liquid crystals (*38*). Thus, confinement-induced chirality can be a general phenomenon for achiral rodlike objects. After reviewing reports on lyotropic liquid crystals that exhibit confinement-induced chirality, we found that their *K*_22_ is at least five times smaller than their *K*_11_ (*31*, *32*). We anticipate that our work will stimulate further studies on achiral lyotropic systems, including conjugated polymers (*24*), supramolecular polymers (*69*), inorganic colloids (*70*, *71*), and filamentous viruses (*21*). Applications of confinement-induced chiral structures can also be explored. For example, confinement of conjugated polymers in droplets, capillaries, or films may lead to spontaneously formed chiral structures with applications in optics and electronics (*24*, *72*). Confinement-induced chirality can also be explored for inorganic colloids, with potential applications in composite materials (*1*), chiral plasmonics (*73*, *74*), and circularly polarized luminescence (*25*, *75*).

In summary, we show that kinetically arrested phase separation of the rigid-rod polymer solutions in narrow capillaries resulted in a scale-free pattern of alternating segments of isotropic and nematic phases. As a result of the interplay of the large elastic anisotropy of the polymer, spatial confinement, surface anchoring, and orientational wetting, the nematic segments acquired chirality by a spontaneously twisted director field, with the fractions of right- and left-handed twisted configurations being approximately equal. Orientational wetting on the curved glass surface by the polymer rods indicated that surface anchoring is sensitive to the local curvature. These findings not only suggest a strategy to fabricate chiral structures from achiral polymers but also provide a mechanistic insight into the chiral self-organization in living organisms and man-made materials under confinement, where polymer elastic anisotropy may play an important role in the emergence of structural chirality.

## MATERIALS AND METHODS

### Materials

2,2-Benzidinedisulfonic acid (BDSA; ≥80.0%), terephthaloyl chloride (TCL; ≥99.0%), 3-aminobenzene-1-sulfonic acid (≥99.0%), sodium hydroxide (NaOH; ≥97.0%), sodium bicarbonate (NaHCO_3_; ≥99.7%), ethanol (≥95.0%), chloroform (CHCl_3_; ≥99.8%), and acetone (≥99.5%) were purchased from Thermo Fisher Scientific. Poly(ethylene glycol) with an average molecular weight of 350 g/mol (PEG-350) was purchased from Sigma-Aldrich. BDSA was neutralized with NaOH to obtain the sodium salt of BDSA (BDSA-Na), which was then purified by reprecipitation in a mixed solvent of deionized water and ethanol. All other reagents were used as received.

### Synthesis and characterization of PBDT

BDSA-Na (5.00 g, 12.9 mmol), NaHCO_3_ (2.38 g, 28.3 mmol), PEG-350 (4.12 g), deionized water (500 ml), and CHCl_3_ (180 ml) were added to a 2-liter Erlenmeyer flask that was placed in an ice bath. The mixture was emulsified by mechanical stirring (1000 rpm) for 10 min. TCL (2.61 g, 12.9 mmol) was dissolved in CHCl_3_ (180 ml) and added to the emulsion in one portion to initiate the interfacial polycondensation reaction. While the flask was maintained in the ice bath, the reaction proceeded under mechanical stirring (1600 rpm) for 30 min until the reaction mixture became highly viscous. The flask was then removed from the ice bath, and the reaction mixture was stirred at 1000 rpm for another 24 hours. Next, the mixture was transferred into a 2-liter single-neck round-bottom flask to remove CHCl_3_ under vacuum. By adding acetone (1.6 liters) to the mixture, the polymer product was precipitated as a gelatinous solid and dried under vacuum. To remove the excessive sodium salt, we redissolved the crude polymer product in deionized water (400 ml) and precipitated it in acetone (1.6 liters). This process was repeated three times, until the pH of the aqueous polymer solution was reduced from 9.0 to 7.0. The PBDT solution was then dried under vacuum to yield 5.4 g of white solid sample (81%). The sample was dissolved in a mixed solvent of D_2_O and CD_3_CN (1:1 w/w) at a concentration of 0.5 wt %, and the proton nuclear magnetic resonance (^1^H NMR) spectrum was collected on a 400-MHz Bruker Avance III NMR spectrometer at 25°C. The peaks in the ^1^H NMR spectrum were assigned to the aromatic protons on the backbone of PBDT (fig. S26). Circular dichroism (CD) spectra of the 0.001 wt % PBDT solution were recorded on a JASCO J-1100 CD spectrometer at 24°C. Figure S27A shows that PBDT is optically inactive (achiral). The monomer BDSA-Na is also achiral (fig. S27B).

The absorption spectrum of BDSA-Na is similar to that of sodium 3-aminobenzenesulfonate (fig. S27, B and C), which suggests that the two phenyl chromophores of BDSA-Na are not strongly conjugated. This is because the sulfonate groups at the ortho positions impose a large steric hindrance that forces the two phenyl rings to be arranged in a nonplanar conformation. Such nonplanar biphenyl conformation induces the axial chirality in BDSA-Na. However, as the CD spectrum shows no CD signal, BDSA-Na must exist as a racemic mixture. On the basis of these results, we ruled out the possibility of PBDT having any chiral preference.

### Fractionation of PBDT

The PBDT sample (5.4 g) was dissolved in deionized water (500 ml) and filtered through a fritted glass disk. This solution was concentrated to *C*_P_ = 7.0 wt % under vacuum. By diluting the 7 wt % PBDT solution with deionized water, a series of solutions of lower concentrations were obtained, which, after 14 days, phase separated into a nematic phase and an isotropic phase. Because unfractionated PBDT synthesized by polycondensation has a broad molecular weight distribution, the phase separation occurred in a broad concentration range of 1.4 to 5.5 wt %, with the volume fraction of the nematic phase increasing nonlinearly with *C*_P_ (fig. S3). To separate the low–molecular weight polymer fraction, we allowed a 2.4 wt % PBDT solution to phase separate into an upper isotropic phase (30% v/v) and a bottom nematic phase (70% v/v). The upper phase containing the low–molecular weight polymer was removed, leaving the bottom nematic phase with *C*_P_ = 2.5 wt %. Upon dilution, the fractionated PBDT solution exhibited a narrower NI coexistence concentration range of 1.3 to 2.5 wt % (figs. S2 and S3).

### Confinement of PBDT solution in capillaries

Cylindrical glass capillary (Drummond Scientific) with an inner diameter of 100, 141, 282, or 400 μm was connected to a 250-μl glass syringe using polytetrafluoroethylene tubing with an inner diameter varying from 305 to 787 μm. After vortex mixing for 30 s, a PBDT solution of a particular concentration was introduced into the capillary by applying negative pressure (syringe withdrawing), and the ends of the capillary were immediately sealed with epoxy glue. The capillaries filled with PBDT solutions were placed horizontally at room temperature (21°C) for 7 days to allow the nematic segment to reach the equilibrium length (fig. S6). No special precautions were taken to suppress vibrations or any other forms of mechanical perturbation. During POM imaging, the chiral nematic segments were sufficiently robust to resist any mechanical perturbations. They remained stable even when the capillaries were placed vertically (fig. S7). Gravity-induced sedimentation of the nematic segments was not observed. Further increase in the capillary diameter resulted in incomplete phase separation, that is, isotropic droplets trapped in nematic segment. Thus, we limited the range of capillary diameter to 100 to 400 μm.

### Hydrophobic modification of the inner surface of the glass capillary

Glass capillaries were washed with deionized water and acetone and subsequently dried in a vacuum oven at 80°C for 24 hours. A plasma cleaner (Harrick Plasma) was then used to clean the capillaries at 300 mtorr for 10 min. Then, the capillaries were filled with trichloro(1*H*,1*H*,2*H*,2*H*-perfluorooctyl)silane solution (1 mM) in hexane for 24 hours. Subsequently to that, the capillaries were washed three times with hexane and acetone and dried with compressed air. In the hydrophobically modified capillary, the wetting angle of water was ~90°, while in the untreated capillary, a wetting angle of ~48° was measured (fig. S20).

### Characterization techniques

A Hitachi HT-7700 microscope was used to perform TEM imaging of PBDT rods at an accelerating voltage of 100 kV. The images were analyzed using ImageJ software (National Institutes of Health). To prepare the sample for imaging, we dispensed a droplet of 5 μl of 0.01 wt % PBDT solution on a carbon-coated lacey grid (Electron Microscopy Sciences). After 1 min, the excessive PBDT solution was absorbed by filter paper, leaving a thin liquid film on the grid. Next, a droplet of 5 μl of 1.0 wt % uranyl acetate solution was dispensed on the grid and left for 1 min. The excessive solution was then absorbed by filter paper, and the sample was allowed to dry in the air in the dark.

Scanning electron microscopy (SEM) imaging of the inner surface of the capillary tube was performed on an FEI Quanta FEG-250 SEM at an accelerating voltage of 10 kV. A 100-μm-diameter capillary tube was fractured to expose its inner surface. The capillary was fixed to the specimen mount using conductive carbon tape.

A polarizing microscope (Olympus BX51) equipped with an eyepiece camera (Swift 10 MP) was used to perform POM imaging of capillary-confined PBDT solutions. The images were analyzed and processed using ImageJ software. The capillaries were fixed onto a glass slide and immersed in glycerol (*n* = 1.470) to reduce light refraction at the glass-air interface. A piece of cover glass was placed on top of the capillaries to obtain a flat layer of glycerol. The samples were placed between the polarizer and analyzer and illuminated using a halogen lamp coupled with a light-balancing daylight filter. Using a Berek compensator (U-CBE), the birefringence (Δ*n*) of the nematic phase of the 1.7 wt % PBDT solution was determined to be 0.004. To determine director orientation, a full-wave plate (U-TP530; λ = 530 nm) was inserted between the sample and the analyzer at an angle of 45° with respect to the crossed polarizer and analyzer.

### Numerical simulations

Numerical simulations were performed to investigate the director alignment in several types of twisted segments, that is, the right- and left-handed segments or racemic-type segments. The computations were carried out using self-built MATLAB programs based on the minimization of the total Landau–de Gennes free energy describing a continuum of liquid crystal

$$F_{\text{total}}=\int f_{\text{bulk}}dr^{3}+\int f_{\text{surf}}dr^{2}$$ (1)

where the bulk energy density is integrated over the three-dimensional volume occupied by the nematic segment. The surface contribution of the free energy comes from the inner surface of the capillary and the NI interfaces, which enclose the nematic segment.

In the **Q**-tensor representation, the bulk free energy has the form (*76*)

$$\begin{array}{c} f_{\text{bulk}}=\frac{A}{2}Q_{\text{ij}}Q_{\text{ji}}+\frac{B}{3}Q_{\text{ij}}Q_{\text{jk}}Q_{\text{ki}}+\frac{C}{4}{\left(Q_{\text{ij}}Q_{\text{ji}}\right)}^{2}\\ \;+\frac{L_{1}}{2}{\left(\frac{\partial Q_{\text{ij}}}{\partial r_{k}}\right)}^{2}+\frac{L_{1}}{2}\frac{\partial Q_{\text{ij}}}{\partial r_{j}}\frac{\partial Q_{\text{ik}}}{\partial r_{k}} \end{array}$$ (2)

with summation over all indices assumed. The molecular tensor order parameter is defined as a three-by-three symmetric, traceless tensor **Q** = *S*/2(3**n** ⊗ **n** − **I**), with the head-tail symmetry of molecular director **n** taken into consideration. Here, *S* is the scalar order parameter, and **I** is the second rank unit tensor.

The bulk free energy density consists of two main parts, of which the first three terms describe the NI transition of the liquid crystal, and the last two terms with spatial derivatives representing the free energy cost of director field deformations. Taking the typical values for modeling thermotropic nematic crystals, we set *A* = −1.72 × 10^5^ J/m^3^, *B* = −2.12 × 10^6^ J/m^3^, and *C* = 1.73 × 10^6^ J/m^3^, which give the equilibrium scalar order parameter *S*_eq_ = 0.533 (*76*). The isosurface of *S* = 0.4 was used to represent the defect at the center of the NI interface. To account for the elastic anisotropic properties that are typical for rigid-rod polymers, we assumed the elasticity parameters *L*_1_ = 0.47 pN and *L*_2_ = 14.7 pN, representing the material with low twisting constraints: *K*_11_/*K*_22_ = *K*_33_/*K*_22_ = 16.7. With *S*_eq_ = 0.533, we converted the elasticity parameters to the elastic constants and found that *K*_11_ = *K*_33_ = 10 pN and *K*_22_ = 0.6 pN (*76*). Saddle-splay elasticity, represented by an additional free energy term with *K*_24_, is known to induce chiral structures in capillary-confined chromonic liquid crystals with a relatively large *K*_24_ (*34*, *35*, *67*). In contrast, we observed a uniaxial director configuration for the confined fully nematic phase of PBDT (fig. S18A), which suggests that *K*_24_ of PBDT is smaller than *K*_22_ (*K*_22_ − *K*_24_ > 0) (*67*). Thus, in this study, the contribution of saddle-splay elasticity to the elastic free energy was ignored.

The surface energy has the form (*77*)

$$f_{\text{surf}}=W\left(1-{\text{sin}}^{4}\theta \right)$$ (3)

where θ is the angle formed by the nematic director and the surface normal direction, and *W* is the anchoring strength coefficient. In our model, we used Eq. 3 to represent the planar anchoring condition at both the NI interface and the NS interface. We also did not apply any azimuthal anchoring potential to the curved glass surface, because the elasticity already forces the surface director orientation to follow the capillary long axis. In the SEM image of the capillary inner surface (fig. S28), we did not observe any grooved structures that could possibly lead to azimuthal anchoring, suggesting that the apparent azimuthal anchoring is governed by elasticity. In addition to the effect of elasticity, we argue that the anisotropic excluded volume interactions between rigid rods and a curved surface do in fact favor azimuthal anchoring along the direction with the lowest curvature, albeit that the effect is quite weak (section S6).

For simplicity, the NI interface was assumed to be hemispherical. For the NS interface, we assumed a surface anchoring strength of *W*_NS_ = 10^−4^ J/m^2^ (*73*), while at the NI interface, *W*_NI_ = 10^−7^ J/m^2^ was adopted (*57*). Such a large difference between the anchoring strength of a solid surface and that of a fluidic NI interface seems to be common for lyotropic liquid crystals of rodlike particles (*58*).

We performed the computation of the Landau–de Gennes free energy using a finite-difference method with equidistant grid spacing in all dimensions. Typically, the computational volume was a box with grid resolution 100 × 100 × 200. The numerical minimization of Landau–de Gennes free energy was followed by an analysis and visualization of the director field using the handedness parameter H . First, the director **n** was extracted from the tensor order parameter **Q** by identifying the largest eigenvalue. The handedness parameter representing the handedness and magnitude of the local twist deformation was given by H  = −**n**·(∇ × **n**) before rescaling, where **n** is the nematic director. Visualization was performed in ParaView (open-source freeware obtained from Kitware, Inc.) with slice planes and directors being colored according to the local value of the handedness parameter. The maximum value of H  found in numerical simulation was 0.4 μm^−1^, and the range for plotting was rescaled to ±0.1 μm^−1^ for clarity.

To compare the numerical findings with experimental results, POM images were generated computationally with a Jones-matrix approach using an in-house–built MATLAB program. First, the Jones matrix for each grid point was calculated using the optical axis that aligned with the local molecular director **n** and the ordinary/extraordinary phase retardation based on the birefringence of the PBDT solution (Δ*n* = 0.004). Images were then constructed by successive multiplication of the Jones matrices across the slice planes perpendicular to the viewing direction. In this work, we combined independent calculations of 640-, 540-, and 430-nm wavelengths, representing red, green, and blue colors, respectively, with different relative intensities, thereby matching those in the experimental incident light intensity and the sensitivity of instruments.

## Supplementary Materials

### The PDF file includes:

Sections S1 to S6 Figs. S1 to S28 Legend for movie S1 References
