# Supplementary material for: Confinement-induced chirality in phase-separated achiral polymer solutions
Source: Sci Adv. 2025 Jul 9;11(28):eadv6734. doi: 10.1126/sciadv.adv6734 (PMC12239940; doi:10.1126/sciadv.adv6734)
Supplement: Supplementary file 1 — Sections S1 to S6 Figs. S1 to S28 Legend for movie S1 References [file sciadv.adv6734_sm.pdf]

Science Advances
AAAS

Supplementary Materials for

Confinement-induced chirality in phase-separated achiral polymer solutions

Baichuan Kou *et al.*

Corresponding author: Eugenia Kumacheva, eugenia.kumacheva@utoronto.ca;
Ivan I. Smalyukh, ivan.smalyukh@colorado.edu

Sci. Adv. **11**, eadv6734 (2025)
DOI: 10.1126/sciadv.adv6734

The PDF file includes:

Sections S1 to S6
Figs. S1 to S28
Legend for movie S1
References

Other Supplementary Material for this manuscript includes the following:

Movie S1

Section S1. Estimation of the dimensions of PBDT rods.

Figure 1 (B and C) in the main text shows bundles formed by negatively stained PBDT rods. At the tip of the bundles, individual PBDT rods can be discerned as the low-contrast, filamentous regions surrounded by the high-contrast region corresponding to the staining agent. Thus, the diameter of PBDT rods can be measured as the width of the low-contrast region, as shown in fig. S1A. Figure S1 (B to D) shows three representative TEM images and the corresponding enlarged images of PBDT rods embedded in staining agent. Based on the analysis of the transverse dimensions of 22 individual PBDT rods their diameter was found to be 1.1 ± 0.2 nm.

While the diameter of PBDT rods appears to be monodisperse, in agreement with previous reports (*46*), the length of PBDT rods must be polydisperse, as indicated by the broad concentration range (1.3-2.5 wt%) of isotropic-nematic coexistence (*53*). However, the exact distribution of rod lengths could not be directly determined by analyzing TEM images of the rods, since most of the rod tips were buried in the bundles. Moreover, the polydispersity of PBDT could not be assessed by aqueous size exclusion chromatography due to polymer aggregation in the eluent phase, which usually has a high ionic strength that reduces the electrostatic repulsion between the PBDT rods. In the present work, due to the difficulties in disentangling the mean and variance of rod length, which would require a comprehensive mapping of the isotropic-nematic phase diagram to find the concentration of PBDT rods in each phase (*53*), we only estimated the upper and lower bounds of the rod length from the isotropic-nematic transition concentrations at C_P of 1.3 and 2.5 wt% (fig. S2 and S3), respectively, using the Onsager theory for monodispersed rods.

The Onsager theory predicts that for uniformly sized rods with uniform length L_{rod} and diameter D_{rod}, the rod volume fraction φ at the isotropic-nematic transition is (*10*)

$$\varphi \approx 4.5\frac{D_{\text{rod}}}{L_{\text{rod}}} \tag{S1.1}$$

and the rod aspect ratio is

$$\frac{L_{\text{rod}}}{D_{\text{rod}}} \approx \frac{4.5}{\varphi} \tag{S1.2}$$

For charged rods, the electrostatic interactions rescale the rod diameter to an effective diameter D_{eff} (*41*). The rod aspect ratio becomes

$$\frac{L_{\text{rod}}}{D_{\text{rod}}} \approx \left(\frac{D_{\text{rod}}}{D_{\text{eff}}}\right)\frac{4.5}{\varphi} \tag{S1.3}$$

The effective diameter D_{eff} is a combination of D_{rod} and the Debye screening length κ^{-1}:

$$D_{\text{eff}} = D_{\text{rod}} + \alpha\kappa^{-1} \tag{S1.4}$$

with a dimensionless parameter α in the form of (*41*)

$$\alpha = \ln A + C_{\text{E}} + \ln 2 - \frac{1}{2} \tag{S1.5}$$

where $C_E \approx 0.577$ is the Euler's constant, and A can be calculated as

$$A = 2\pi v_{\text{eff}}^{2}\kappa^{-1} l_{\text{B}} e^{-\kappa D_{\text{rod}}} \tag{S1.6}$$

In equation (Eq.) S1.6, v_{eff} is the effective linear charge density, i.e., the effective number of charges per unit length of PBDT rods, $l_B \approx 0.71$ nm is the Bjerrum length in water at 21°C, the

temperature of our experiments. For the salt-free solution of PBDT rods, the Debye screening length takes the form of

$$\kappa^{-1} = \left(8\pi l_{\mathrm{B}} N_{\mathrm{A}} I\right)^{-1/2} \tag{S1.7}$$

where $N_A = 6.02 \times 10^{23}$ mol^{-1} is the Avogadro constant and I is the ionic strength of the salt-free solution. Once ν_{eff} and I are determined, the effective diameter and thus the aspect ratio of PBDT rods can be obtained.

Based on the PBDT structure, the average distance between the charged groups on the rod surface is $l = 0.42$ nm (*46*). The Manning parameter q for counterion condensation is (*52*)

$$q = \frac{l_{\mathrm{B}}}{l} \approx 1.69 > 1 \tag{S1.8}$$

The larger than unity Manning parameter suggests that a fraction of the counterions (Na^+) are condensed onto the negatively charged PBDT rods, which reduces the effective linear charge density to a universal value of

$$\nu_{\mathrm{eff}} = \frac{1}{l_{\mathrm{B}}} \approx 1.40 \text{ nm}^{-1} \tag{S1.9}$$

The fraction of the disassociated counterions is $q^{-1} \approx 0.59$. Thus, the value of I can be calculated from the polymer concentration C_P (wt%), the density of PBDT solution ($\rho_{sol} \approx 1.0$ g/cm^3), and the molecular weight of the repeating unit of PBDT ($M = 518$ g/mol) as

$$I = \frac{1}{2}\sum_i C_i Z_i^2 = \frac{2C_{\mathrm{P}} q^{-1}}{\rho_{\mathrm{sol}} M} \tag{S1.10}$$

where C_i is the molar concentration of the ion species i and $Z_i = 1$ is the charge number of the ion. We note that, for simplicity, both the $-SO_3^-$ groups of PBDT rods and the disassociated counterion Na^+ were regarded as monovalent free ions contributing to the ionic strength of the salt-free solution. A more rigorous treatment of the Debye screening length in polyelectrolyte systems can be found elsewhere (*78*).

By substituting ν_{eff} and I in Eqs. S1.6 and S1.7 with their calculated values, we found the effective diameters of PBDT rods at C_P of 1.3 wt% and 2.5 wt% to be 6.2 nm and 4.1 nm, respectively. The effective diameters in the salt-free solutions of PBDT rods are approximately five times larger than the 1.1 nm hard rod diameter measured from TEM images, indicating a substantial contribution of electrostatic repulsion to the formation of nematic phase at a low polymer concentration.

To calculate the rod aspect ratio, we converted the weight concentration of PBDT rods into the rod volume fraction φ, using the density of PBDT ($\rho_P \approx 1.4$ g/cm^3) (*46*) and the density of the solution ($\rho_{sol} \approx 1.0$ g/cm^3):

$$\varphi = C_{\mathrm{P}} \frac{\rho_{\mathrm{sol}}}{\rho_{\mathrm{P}}} \tag{S1.11}$$

By substituting D_{eff} and φ in Eq. S1.3 with their calculated values, we found the upper and lower bounds of rod aspect ratio to be 86 and 68, respectively. Thus, the length of PBDT rods is estimated to be in the range of 75-94 nm, much shorter than the length of 250-500 nm given by Eq. S1.2, in which the electrostatic interactions are ignored.

The estimated length of PBDT rods is also shorter than the typical length of the bundles observed in TEM images. On one hand, this can be explained by the fact that the bundles are formed by multiple rods. On the other hand, we admit that our estimation based on the Onsager theory is only semiquantitative, and a rigorous theory to describe the phase behavior of rodlike polyelectrolytes in their salt-free solutions is still lacking.

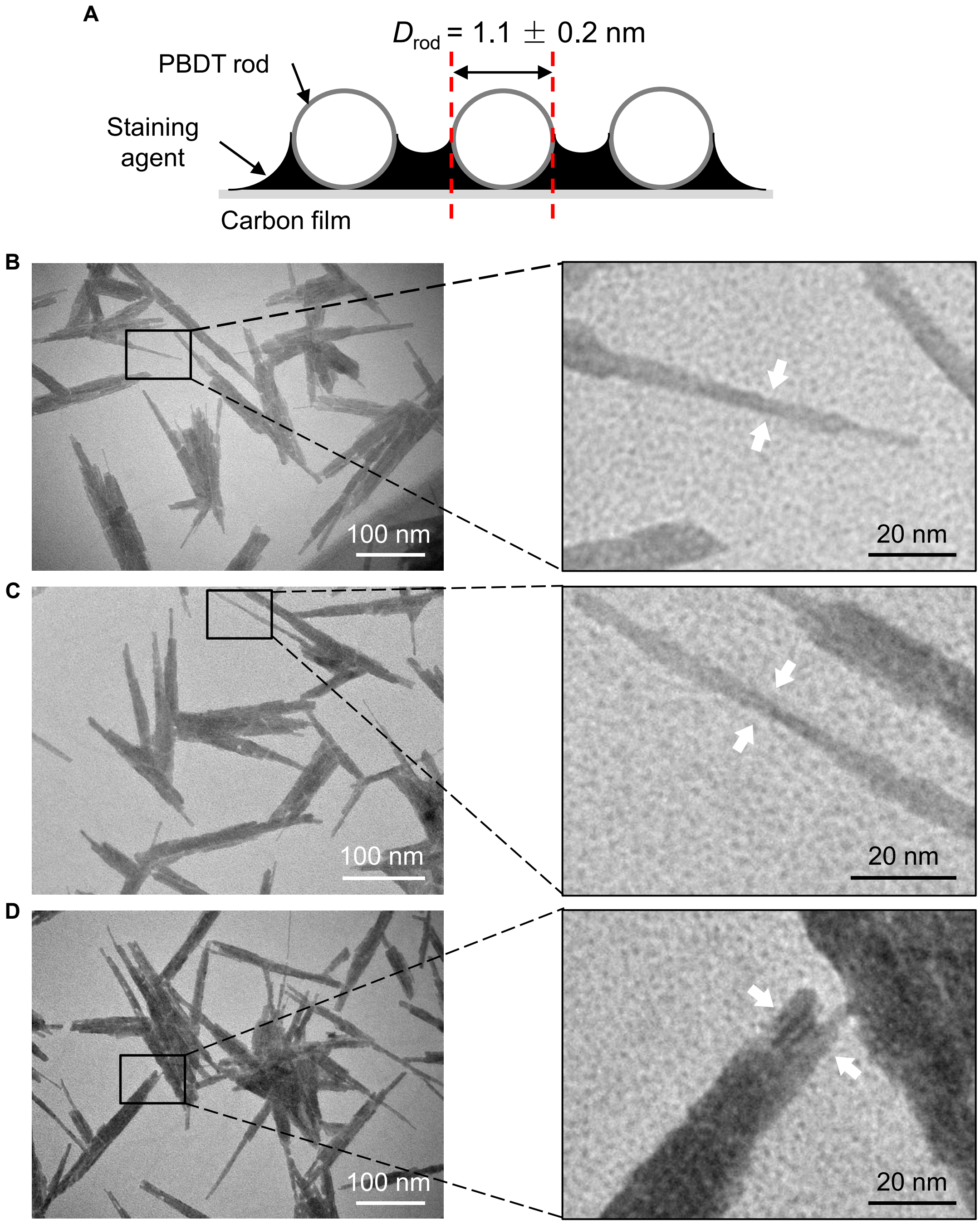

Fig. S1. Measurement of the diameter of PBDT rods. (**A**) Schematic of the transverse cross-section of the bundle formed by negatively stained PBDT rods. (**B**) to (**D**) Representative TEM images used to determine the diameter of PBDT rods. The enlarged images in the right column show individual PBDT rods at the tip of the bundles.

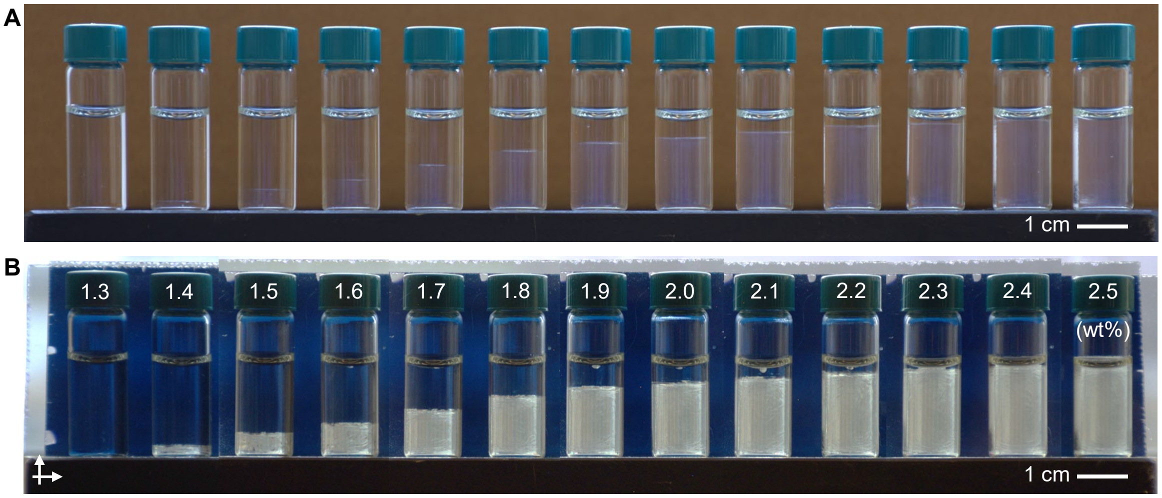

Fig. S2. Photographs of phase-separated PBDT solutions after two-week storage at 21°C. (**A**) The photograph (taken without crossed polarizers) shows the coexistence of the transparent isotropic phase (top) and the translucent nematic phase (bottom). (**B**) The photograph taken with crossed-polarizers shows the dark isotropic phase and the birefringent nematic phase. The numbers in (B) are the mass concentrations (C_P) of the PBDT solutions.

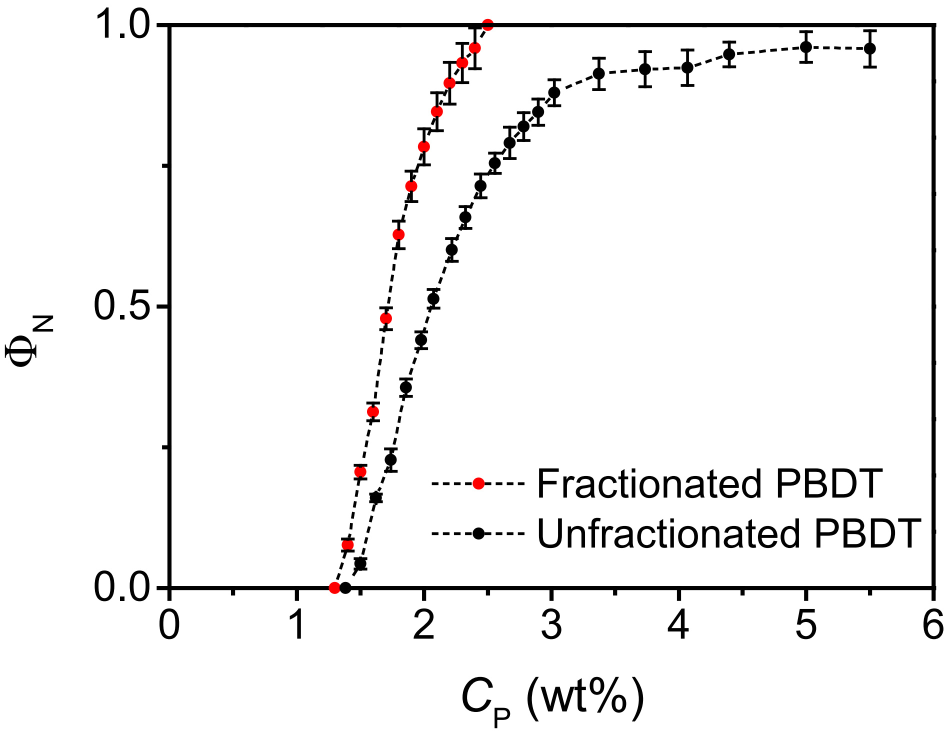

Fig. S3. Variation in the volume fraction of the nematic phase with increasing PBDT concentration. The volume fractions were determined by measuring the relative height of the nematic phase in fig. S2. The error bars are the standard deviations of Φ_N from 5 measurements.

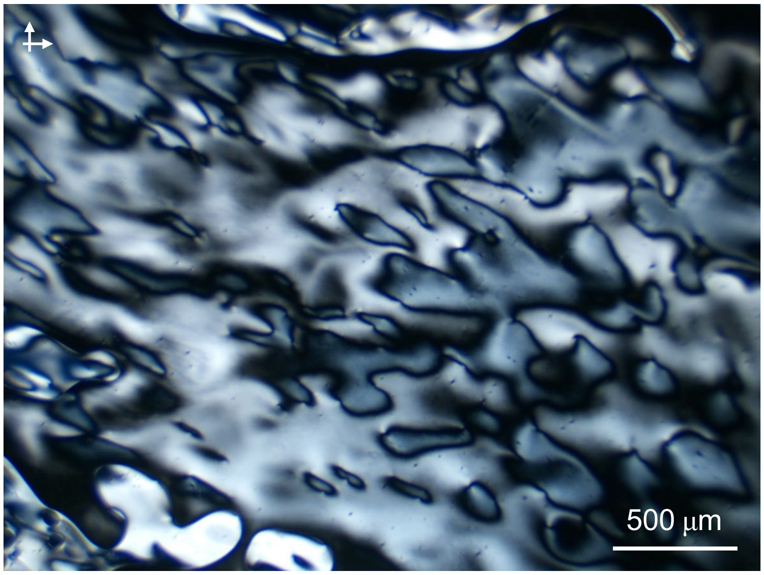

Fig. S4. POM image of the nematic phase of the 1.7 wt% PBDT solution.

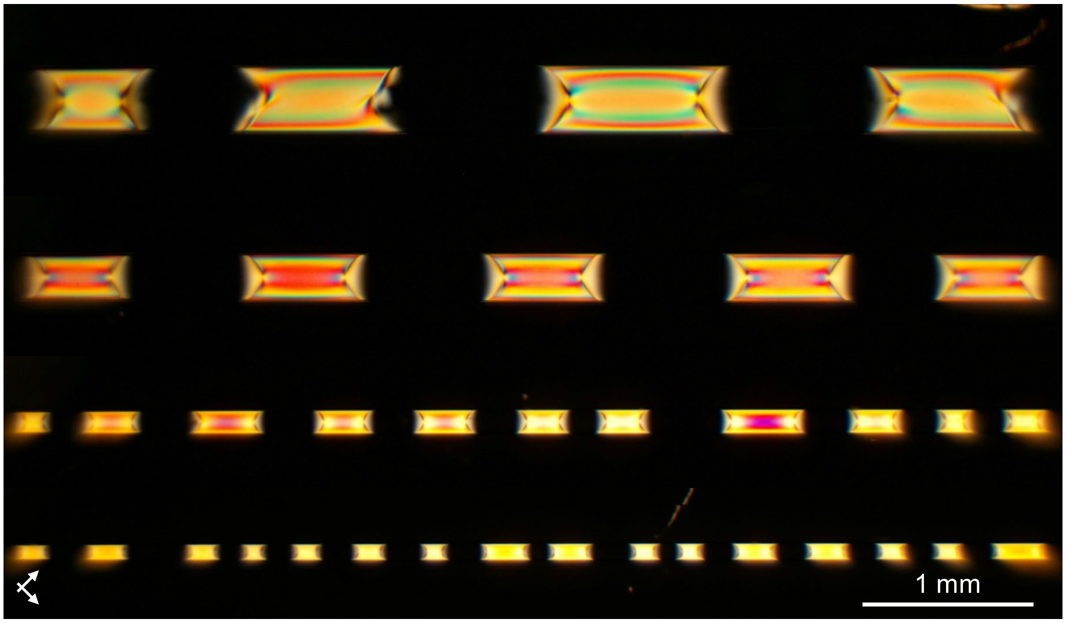

Fig. S5. POM images of PBDT solutions confined to capillaries with different diameters. Capillary diameter D_{cap} (top to bottom) was 400, 282, 141, and 100 μm. The capillaries were aligned 45° to the crossed polarizer and analyzer. C_P = 1.7 wt%.

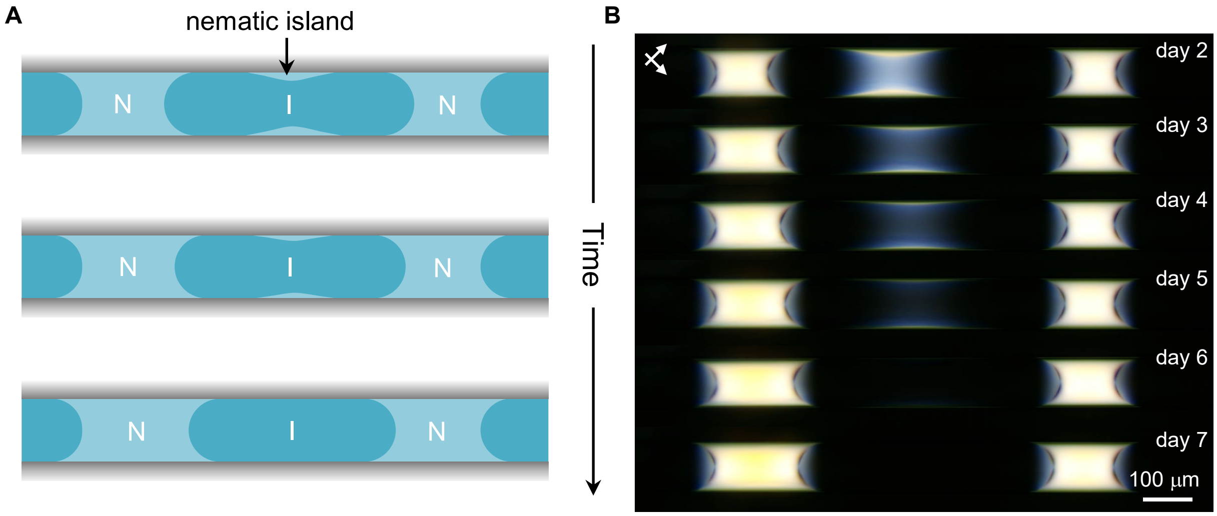

Fig. S6. Slow dissolution of nematic island. (**A**) Schematic of the dissolution of a nematic island formed in the isotropic phase between two nematic segments. (**B**) Time-lapse POM images showing the disappearance of the nematic island and the corresponding increase in the lengths of the two neighboring nematic segments after 7-day equilibration at 21°C. C_P = 1.6 wt%. D_{cap}= 100 μm.

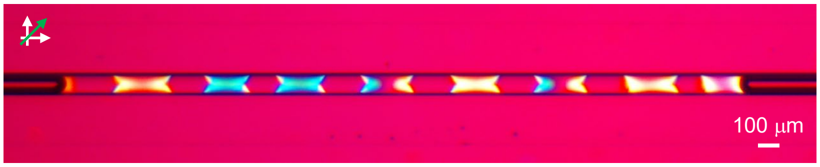

Fig. S7. POM image of the confined phase-separated PBDT solution stored vertically. The sample was stored vertically for 7 days at 21°C. C_P = 1.7 wt%. D_{cap} = 100 μm. The length of the solution slug was 3.2 mm.

Section S2. Determination of the volume fraction of the nematic phase in the capillaries.

Figure. S8 shows the geometry of confined nematic and isotropic segments. This geometry was used to determine the volume fraction of the nematic phase, Φ_N, in the capillaries.

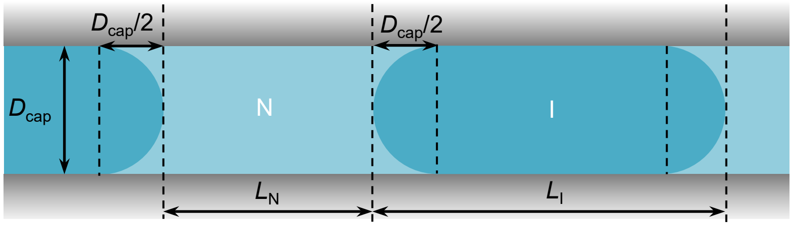

Fig. S8. Schematic of the geometry of capillary-confined nematic and isotopic segments.

By assuming that the meniscus of the nematic segments has a perfect hemi-spherical shape, the volume of a nematic segment, v_N, was determined as

$$v_N = \pi\left(\frac{D_{cap}}{2}\right)^2 L_N + \left[\pi\left(\frac{D_{cap}}{2}\right)^2 D_{cap} - \frac{4}{3}\pi\left(\frac{D_{cap}}{2}\right)^3\right] \quad \text{(S2.1)}$$

where D_{cap} is the inner diameter of the capillary and L_N is the nematic segment length. For a capillary containing an equal number n of nematic and isotropic segments, the total volume of the nematic phase, V_N, was found as

$$\begin{aligned} V_N &= \sum_i^n \pi\left(\frac{D_{cap}}{2}\right)^2 (L_N)_i + n\left[\pi\left(\frac{D_{cap}}{2}\right)^2 D_{cap} - \frac{4}{3}\pi\left(\frac{D_{cap}}{2}\right)^3\right] \\ &= n\langle L_N\rangle \pi\left(\frac{D_{cap}}{2}\right)^2 + \frac{n}{3}\pi\left(\frac{D_{cap}}{2}\right)^2 D_{cap} \end{aligned} \quad \text{(S2.2)}$$

Similarly, for the isotropic segments with length L_I, the total volume of the isotropic phase, V_I, was determined as

$$V_I = n\langle L_I\rangle \pi\left(\frac{D_{cap}}{2}\right)^2 - \frac{n}{3}\pi\left(\frac{D_{cap}}{2}\right)^2 D_{cap} \quad \text{(S2.3)}$$

The volume fraction of the nematic phase, Φ_N, was calculated as

$$\Phi_N = \frac{V_N}{V_N + V_I} = \frac{\frac{\langle L_N\rangle}{D_{cap}} + \frac{1}{3}}{\frac{\langle L_N\rangle}{D_{cap}} + \frac{\langle L_I\rangle}{D_{cap}}} \quad \text{(S2.4)}$$

The value of Φ_N in the capillaries was obtained from $\langle L_N\rangle/D_{cap}$ measured in capillaries with different diameters or with different polymer concentrations (figs. S9 to S13). We found that the value of Φ_N in the capillaries was 5-13% smaller than that measured for the macroscopically phase-separated solution in the vial. The assumption about the hemi-spherical meniscus shape and the approximate estimation of Φ_N in macroscopically phase-separated solutions by measuring the relative height of the nematic phase could lead to this difference.

We also ruled out the possibility that the curved nematic/isotropic (NI) interface compressed the nematic segments, resulting in a notably higher polymer concentration of the nematic segments compared to that of the nematic phase in the vial. This is because the Laplace pressure ($\Delta P = 2\gamma_{\mathrm{NI}}/D_{\mathrm{cap}}$) associated with the curved interface is small compared to the osmotic pressure ($P \sim \gamma_{\mathrm{NI}}/L_{\mathrm{rod}}$) at isotropic-nematic coexistence

$$\frac{\Delta P}{P} \sim O\left(\frac{L_{\mathrm{rod}}}{D_{\mathrm{cap}}}\right) \tag{S2.5}$$

where γ_{NI} is the interfacial tension of the NI interface (*57*). Thus, we conclude that confinement did not substantially change the pressure at isotropic-nematic coexistence. The polymer concentration in and the volume fraction of each phase remained the same with those of macroscopically separated phases.

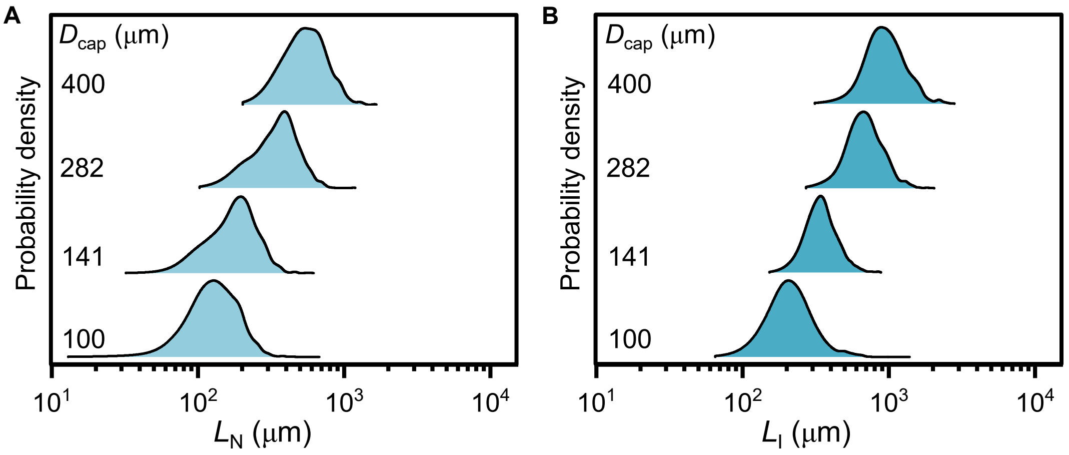

Fig. S9. Distribution of L_N and L_I of PBDT solutions confined to capillaries with different diameters. (**A**) Distribution of L_N. (**B**) Distribution of L_I. For each capillary diameter D_{cap}, the data were obtained from about 500-1000 segments. Capillary diameter D_{cap} (top to bottom) was 400, 282, 141, and 100 μm. C_P = 1.7 wt%.

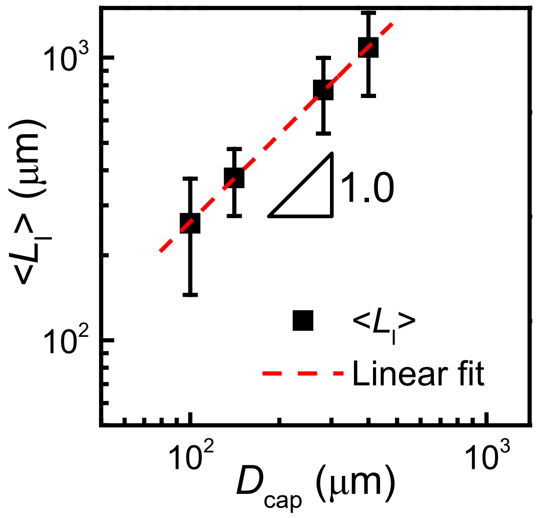

Fig. S10. Scaling relationship between <L_I> and D_{cap}. The dashed red line is the linear fit of the results obtained in capillaries with different diameters. The data were obtained from about 500-1000 nematic segments for each capillary diameter. The error bars represent the standard deviations of L_I. C_P = 1.7 wt%.

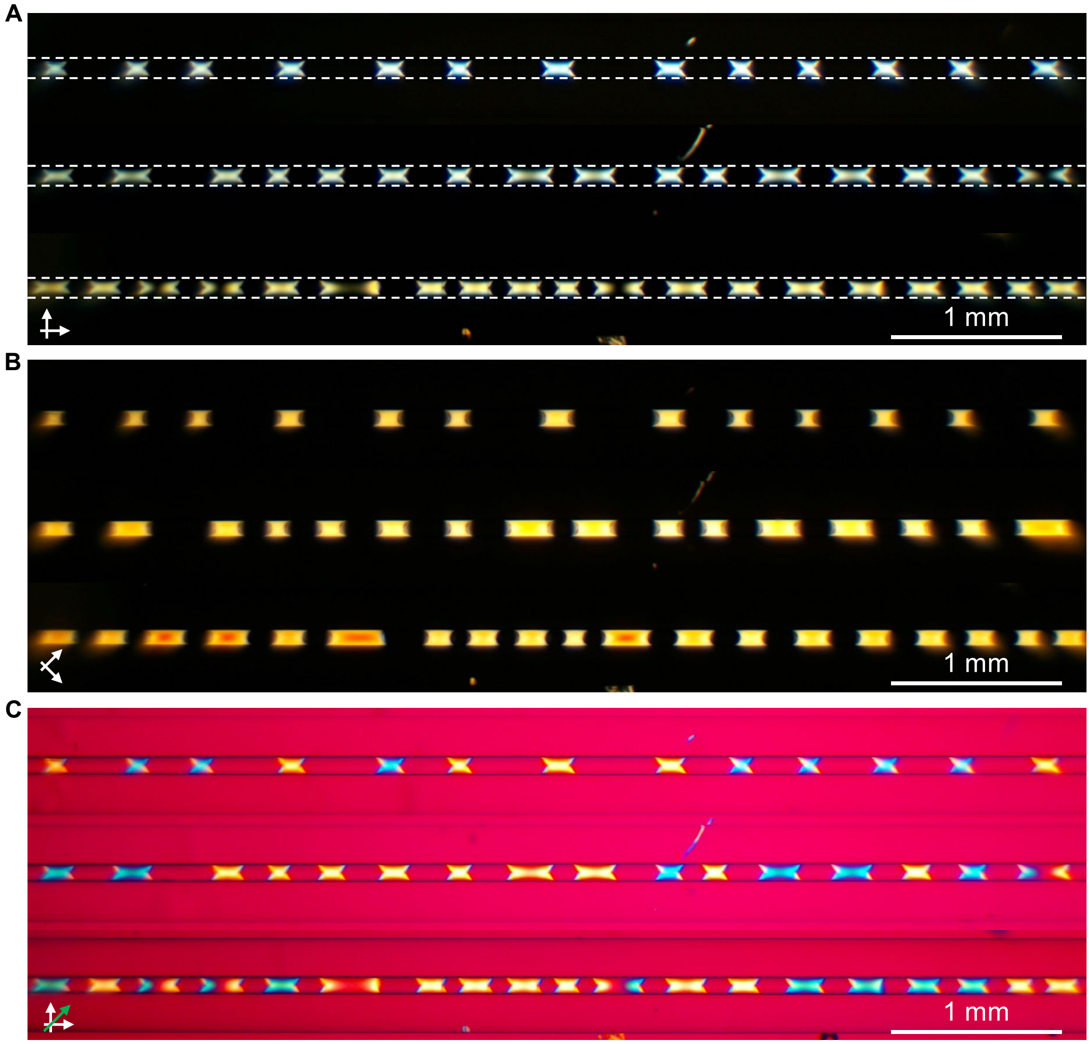

Fig. S11. POM images of PBDT solutions confined to 100 µm-diameter capillaries. (**A**) The capillaries were parallel to the polarizer. The glass surface is marked by dashed white lines. (**B**) The capillaries were rotated by 45°. (**C**) The capillaries were parallel to the polarizer and a full-wave plate was inserted between the sample and the analyzer. The green arrow indicates the slow axis of the full-wave plate. Polymer concentration C_P (top to bottom in each panel) was 1.6, 1.7, and 1.8 wt%.

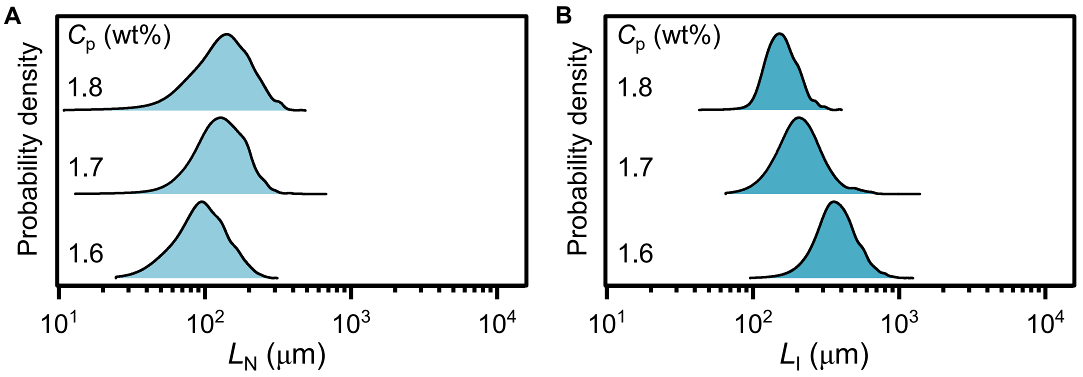

Fig. S12. Distribution of L_N and L_I of PBDT solutions with different concentrations confined to 100 μm-diameter capillaries. (**A**) Distribution of L_N. (**B**) Distribution of L_I. For each concentration, the data were obtained from about 750-1000 segments. Polymer concentration C_P (bottom to top) was 1.6, 1.7, and 1.8 wt%.

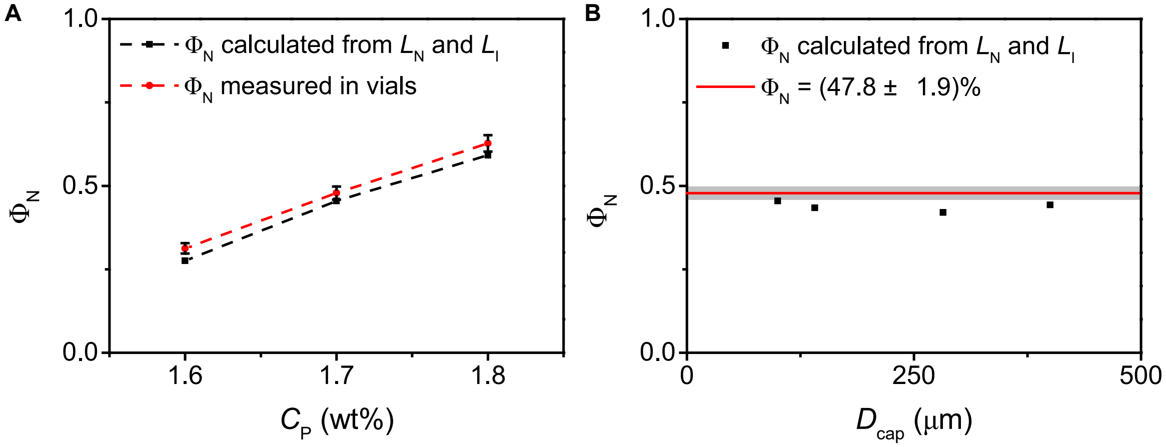

Fig. S13. Volume fractions of the nematic phase confined to the capillaries and in the macroscopically phase-separated PBDT solutions. (**A**) Variation in the volume fraction Φ_N of the nematic phase in 100 μm-diameter capillaries (black squares) plotted *vs*. C_P shows a similar trend with Φ_N measured in macroscopically phase-separated polymer solutions (red dots). (**B**) The values of Φ_N for the PBDT solutions confined to capillaries with different diameters. The solid red line shows the volume fraction of the nematic phase of the macroscopically phase-separated PBDT solution with C_P = 1.7 wt%.

Section S3. Elliptical polarization of light by the nematic segments.

The nematic segments were birefringent, however they did not show complete extinction when rotated between the crossed polarizer and analyzer. This effect indicated that the linearly polarized incident light became elliptically polarized after being transmitted through the nematic segments. By inserting the full-wave plate between the sample and the analyzer, the main body of the nematic segments appeared blue or yellow. These colors corresponded to different twisted orientations of the director field in the nematic segments, which rotated the major axis of the polarization ellipse of the transmitted light in different directions.

To characterize the rotation direction and angle, the 100 µm-diameter capillary was aligned parallel to the analyzer, and the polarizer was rotated in steps of 3.6° to capture a series of POM images at different polarizer-analyzer angles ($\theta_{P\text{-}A}$) (*31*). For a blue segment (L_N/D_{cap} = 1.58) and a yellow segment (L_N/D_{cap} = 1.60), the variation in light intensity at the segment center was plotted as a function of $\theta_{P\text{-}A}$ (fig. S14). The variation in light intensity was fitted to a sine-square function. The lowest intensity was achieved at approximately 80° for the blue segment and at 98° for the yellow segment. Thus, the major axis of the light transmitted through the blue segment and the yellow segment was rotated by 10° and 8°, respectively, to opposite directions.

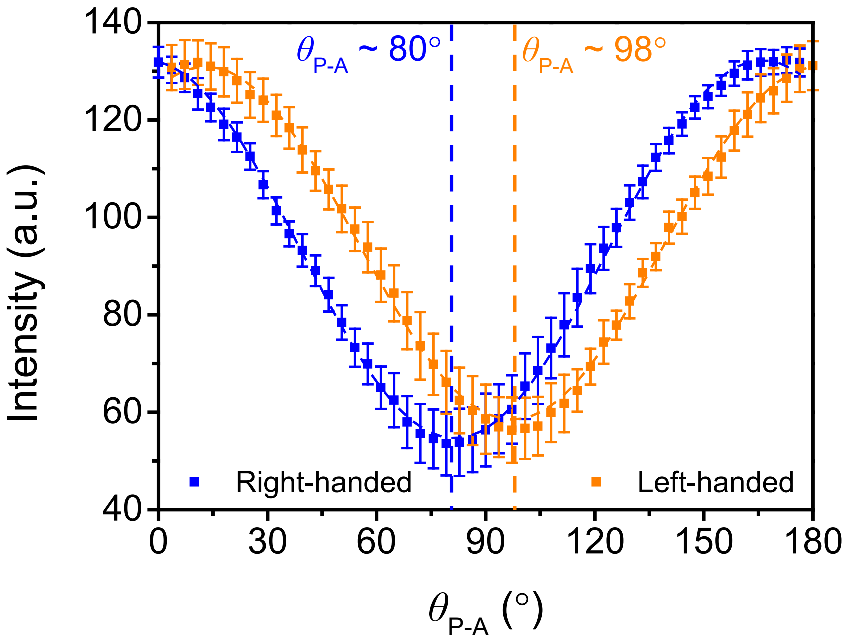

Fig. S14. Variation in the intensity of light measured at the center of a right-handed nematic segment (blue) and a left-handed nematic segment (orange), plotted as a function of the polarizer-analyzer angle ($\theta_{P\text{-}A}$). Error bars are the standard deviations of light intensities measured in the central segment region defined by a rectangle with dimensions (height × width) of 33 µm × L_N/3.

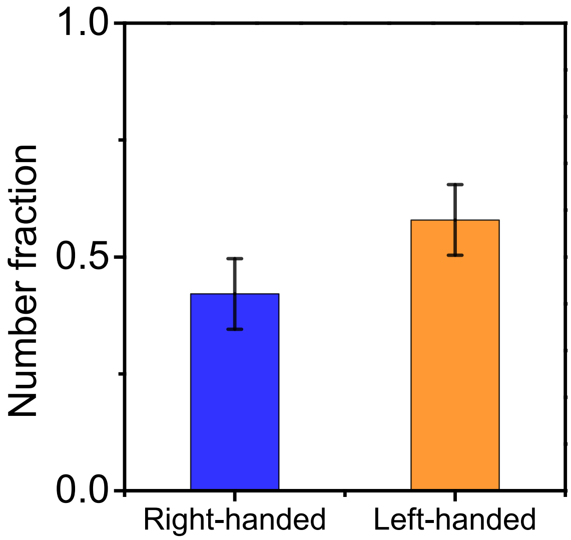

Fig. S15. Number fractions of right- and left-handed chiral nematic segments. The data were obtained from 20 capillaries from about 1000 segments. The error bars are the standard deviations of the number fractions obtained from 20 capillaries. C_P = 1.7 wt%. D_{cap} = 100 µm.

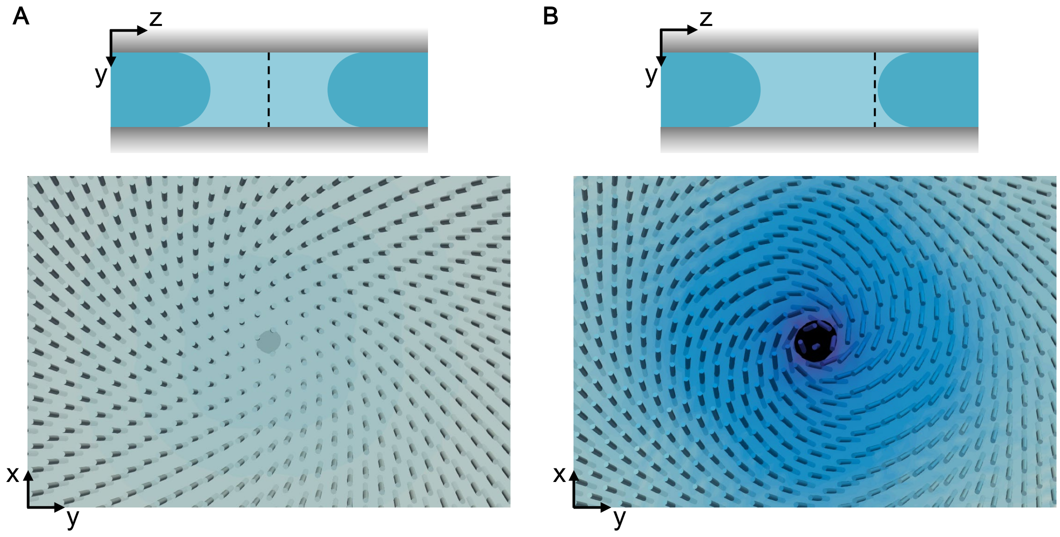

Fig. S16. Enlarged transverse cross-sections of the right-handed chiral nematic segment. (**A**) Transverse cross-section at the midplane of the segment. (**B**) Transverse cross-section near the defect. The dashed black lines in the schematics show the locations of the corresponding transverse cross-sections. The distance from the defect to the cross-section plane in (B) is $0.02D_{\mathrm{cap}}$. $L_{\mathrm{N}} = 1.54D_{\mathrm{cap}}$. $D_{\mathrm{cap}} = 100$ μm.

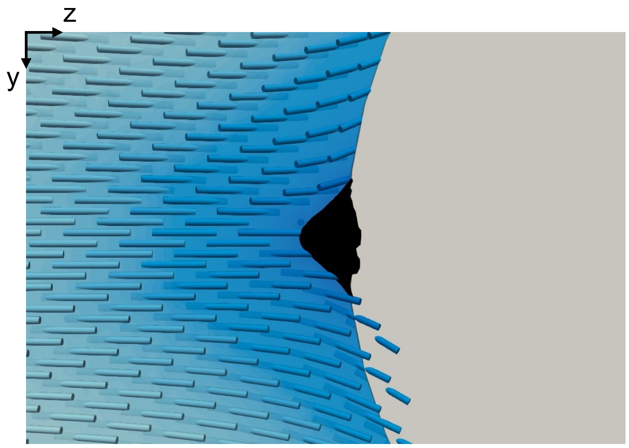

Fig. S17. Enlarged longitudinal cross-section of the right-handed chiral nematic segment showing the director field close to the defect. The black region represents the isosurface of $S = 0.4$.

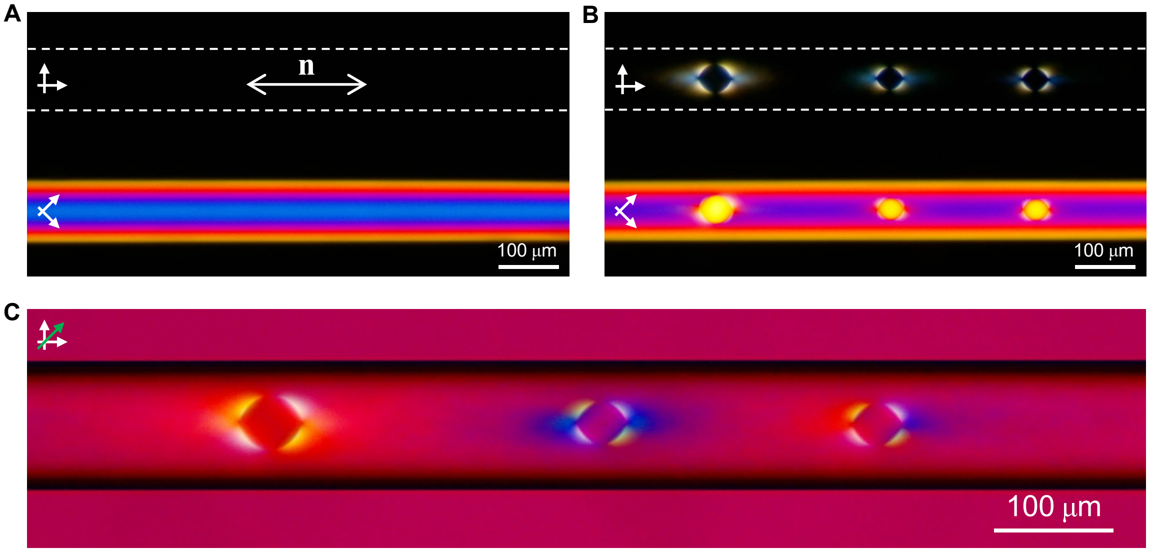

Fig. S18. POM images of capillary-confined 2.5 wt% and 2.4 wt% PBDT solutions. (**A**) The confined fully nematic PBDT solution appeared dark when the capillary was parallel to the polarizer. Bright birefringent colors were observed when the capillary was rotated by 45°. C_P = 2.5 wt%. (**B**) Isotropic droplets formed in the confined PBDT solution showed birefringent tails extending along the capillary long axis. C_P = 2.4 wt%. (**C**) Droplet tails as in (B) showed blue and yellow colors under the full-wave plate as the result of chiral twisted structures (*31*). D_{cap} = 100 µm.

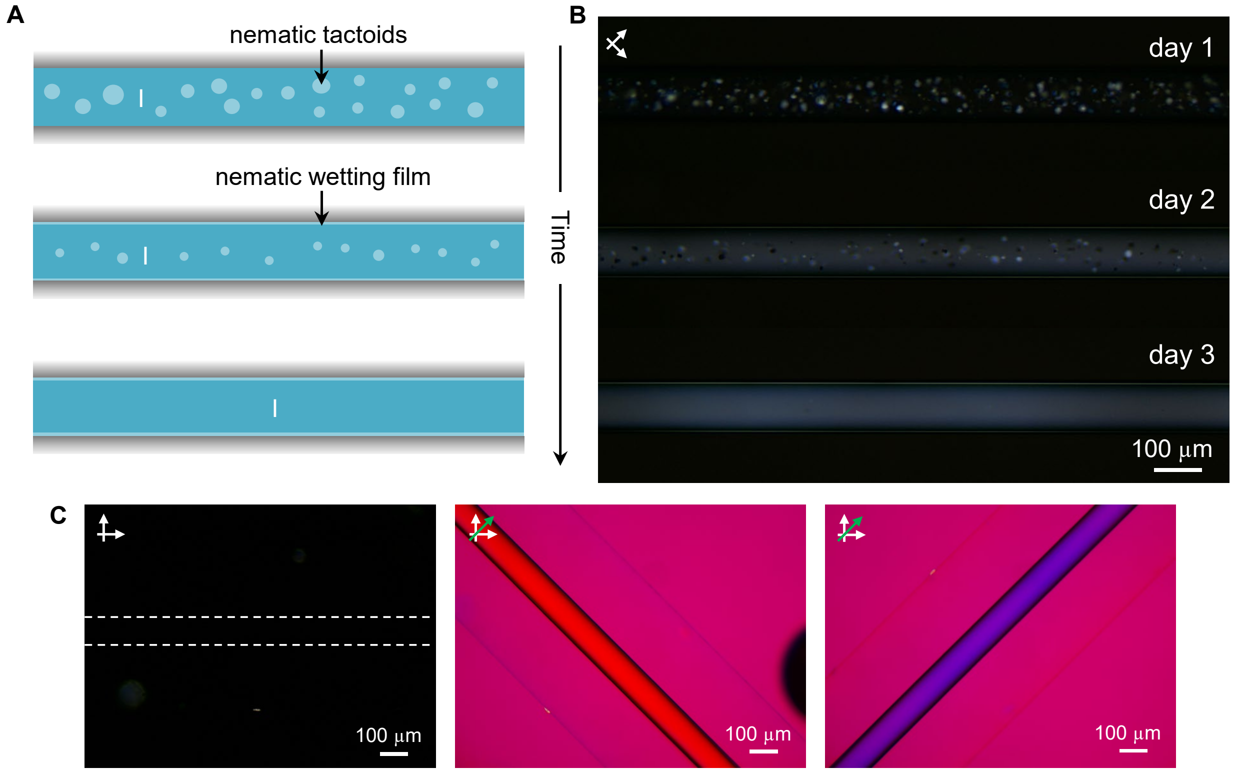

Fig. S19. Orientational wetting in the thin nematic wetting film. (**A**) Schematic of the formation of the thin nematic wetting film. Top-to-bottom: nucleation of tactoids, wetting of the glass surface by the nematic phase, and the complete dissolution of tactoids. (**B**) Top-to-bottom: corresponding time-lapse POM images acquired during the course of 3 days after sample preparation, with the capillary aligned 45° to the polarizer. (**C**) POM images of the same sample shown in the bottom image in (B), with the capillary aligned parallel to the polarizer (left), perpendicular to the slow axis of the full-wave plate (middle), and parallel to the slow axis of the full-wave plate (right). The dashed white lines outline the glass surface. To obtain the thin nematic wetting film, we used a PBDT solution with C_P = 1.4 wt%, which is close to the isotropic-nematic transition concentration. The thin nematic wetting film was formed due to the small volume fraction Φ_N = (7.6 ± 1.0)% of the nematic phase (figs. S2 and S3) and the strong tendency of the nematic phase to wet the glass surface. D_{cap} = 100 μm.

Section S4. Orientational wetting on a hydrophobic surface.

Figure S20 shows that the wetting angle at the water/air/solid contact line is approximately 48° for the untreated capillary. For the hydrophobically modified capillary, the wetting angle increased to approximately 90°, indicating a successful hydrophobic surface modification.

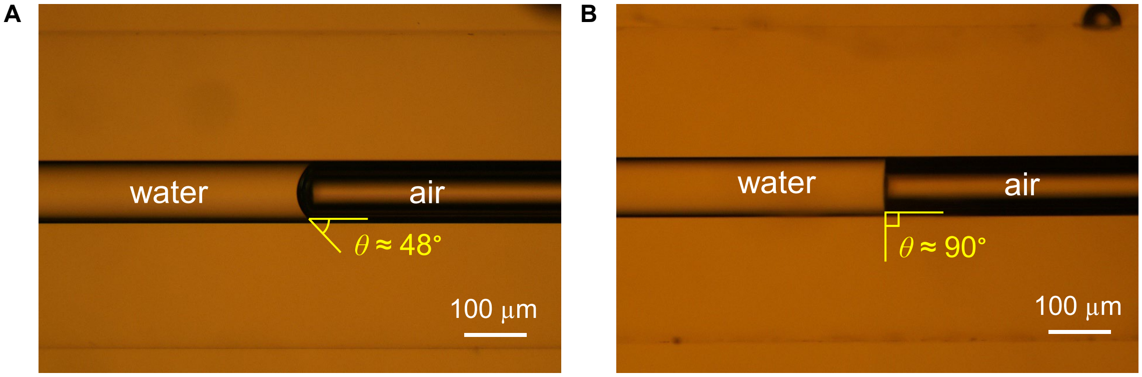

Fig. S20. Comparison of the wetting angles at the water/air/solid contact line in untreated and hydrophobically modified capillaries. (**A**) The wetting angle of water in the untreated capillary is approximately 48°. (**B**) The wetting angle of water in the hydrophobically modified capillary is approximately 90°.

A hydrophobically modified capillary was filled with PBDT solution (C_P = 1.7 wt%) and equilibrated at 21°C for 7 days. The dark appearance of the middle of the segments in fig. S21A indicates a uniaxial orientation of PBDT rods parallel to the capillary long axis. In fig. S21C, the blue and yellow interference colors were observed only near the defect on the NI interface, similar to the chiral structure formed near the confined isotropic droplets (fig. S18, B and C). These observations suggest that in the hydrophobically modified glass capillary the chiral structure of the nematic segments was not as well-defined as that in untreated capillaries.

In contrast to the complete wetting of the hydrophilic surface in untreated capillaries, in the hydrophobically modified capillaries the wetting angle of the menisci of the nematic segments was not zero (fig. S21D). We also observed sessile nematic droplets that only partially wet the glass surface and persisted over 7 days (fig. S21, A to C). The stability of the sessile nematic droplets was in stark contrast to the nematic islands that eventually dissolved to join the isotropic phase (fig. S6). Collectively, these results indicate incomplete wetting of the hydrophobic surface by the nematic phase.

While the effect of the surface energy on the wetting behavior of the nematic phase deserves future investigation, the fact that the nematic phase can wet a hydrophobically modified surface suggested that the wetting behavior of the nematic phase of rigid rods against a hard wall is a generic effect, as predicted by theoretical studies of orientational wetting (*60*). Such equilibrium wetting behavior is mainly driven by the excluded volume interactions between the rigid rods and the hard wall.

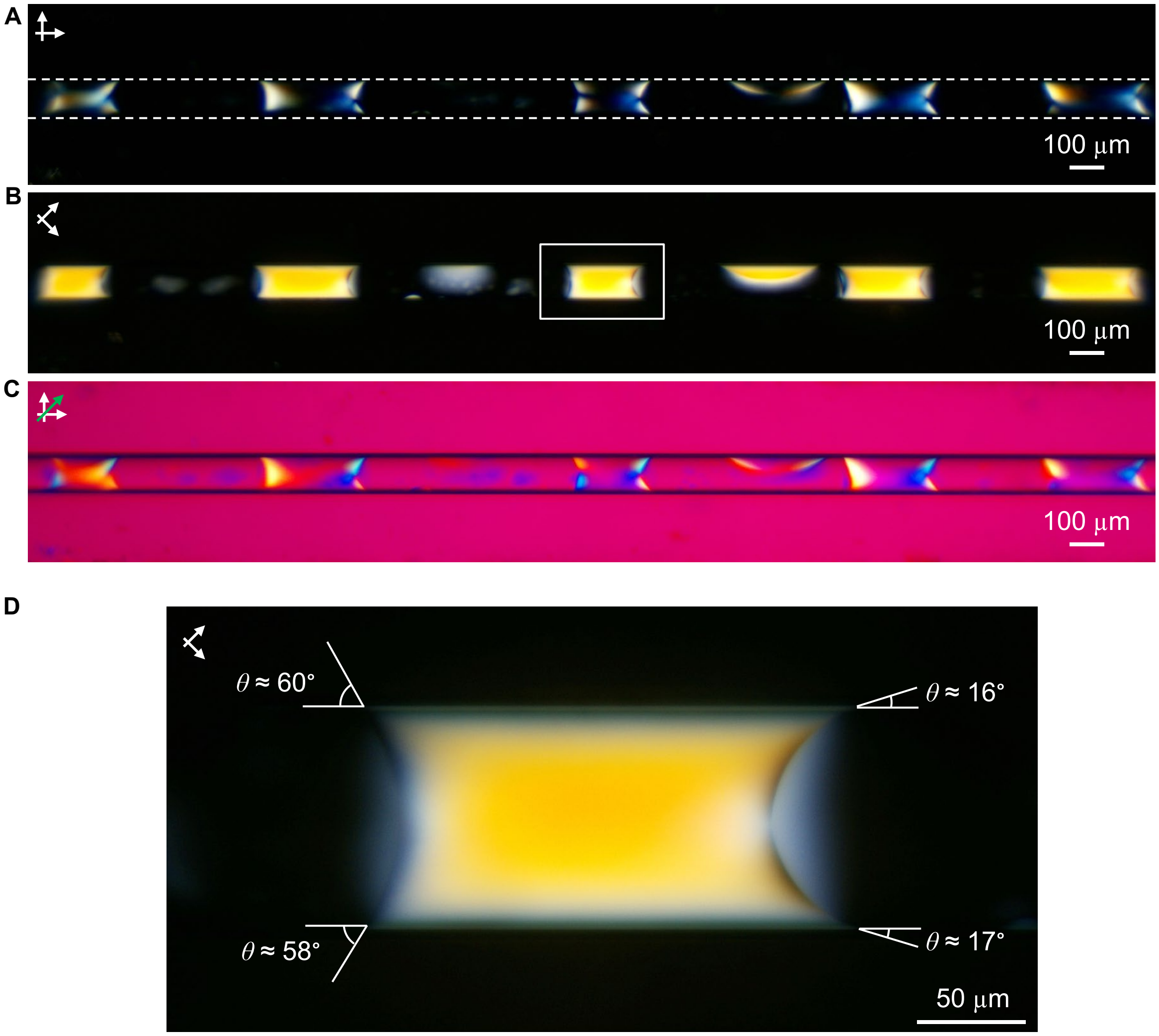

Fig. S21. POM images of the nematic segments formed in a hydrophobically modified capillary. (**A**) The capillary was aligned parallel to the polarizer. The dashed white lines outline the glass surface. (**B**) The capillary was rotated by 45°. (**C**) The capillary was aligned parallel to the polarizer and a full-wave plate was inserted between the capillary and the analyzer. (**D**) Enlarged POM image of the nematic segment marked by the white rectangle in (B). The segment exhibits non-zero wetting angles at the four meniscus tips. C_P = 1.7 wt%. D_{cap}= 100 μm.

Section S5. Characteristic length scale of the meniscus tip with uniform director field.

The characteristic length scale, ξ, is defined as the length of the meniscus tip region within which the director field stays uniform, that is, remains parallel to the glass surface due to the strong polar anchoring strength of the glass surface (fig. S22). Beyond ξ, splay deformation took place, that is, the director orientation at the NI interface became tilted with respect to the glass surface, to reduce the surface anchoring energy. Thus, the value of ξ is determined by the competition between the elastic energy of the director field and the surface anchoring energy of the NI interface.

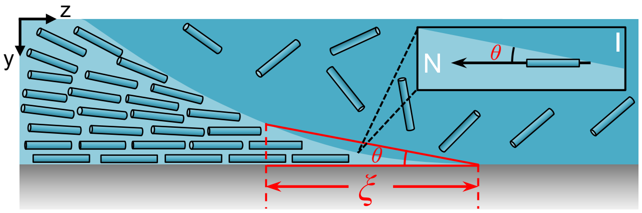

Fig. S22. Schematic of the geometry of the meniscus tip. The transition from a uniform director field to a deformed one takes place at the characteristic length scale ξ.

To determine ξ, we compared a situation where the director field follows both the glass surface and the NI interface, and ends in a surface disclination line, at the cost of an elastic free energy, with that where the director field becomes uniform at the expense of imperfect anchoring. To start with the former, only the contribution of splay deformation to the elastic energy was considered, since the meniscus tip has a wedge-like geometry. Thus, the elastic free energy, F_E, of a meniscus tip of length ξ scales with the splay elastic constant K_{11} and ξ as (*62*)

$$F_E \sim K_{11} D_{cap} \left(\frac{\xi}{D_{cap}} \right) \ln \frac{\xi}{\beta} \sim K_{11} \xi \ln \frac{\xi}{\beta} \qquad \text{(S5.1)}$$

Here we accounted for the length of the surface disclination loop (of order D_{cap}), the fact that only a fraction ξ/D_{cap} of the nematic phase has a uniform director field, and the cut-off length β that avoids the divergence of F_E per unit length at the contact line. The cut-off length is defined as the distance from the contact line within which the splay deformation was replaced by an isotropic phase. The value of β is on the same length scale with the length of PBDT rods (*62*), and thus much smaller than ξ. Note that here, and below, we ignore numerical prefactors that we presume to be of order unity. Hence, we use the symbol ~ rather than the equal or approximately equal sign.

The surface anchoring energy, F_S, associated with the non-planar anchoring of PBDT rods at the NI interface with an anchoring strength of W_{NI}, is expressed by integrating the Rapini-Papoular form of anchoring energy over the NI interface as

$$F_S \sim \int W_{NI} \sin^2 \theta ds \qquad \text{(S5.2)}$$

where θ is the angle between the rods and the surface tangent plane (fig. S22). By assuming that the value of θ is constant for the meniscus tip of length ξ and that ξ is small, compared to the capillary diameter D_{cap}, the value of $\sin\theta$ is

$$\sin\theta \sim \theta \sim \frac{\xi}{D_{\text{cap}}} \tag{S5.3}$$

The surface area of the NI interface with non-planar rod anchoring is approximately $D\xi$, apart from a numerical prefactor, and F_S therefore is

$$F_S \sim W_{NI} \frac{\xi^3}{D_{\text{cap}}} \tag{S5.4}$$

The difference between the surface anchoring energy and the elastic energy is

$$\Delta F = F_S - F_E \sim W_{NI} \frac{\xi^3}{D_{\text{cap}}} - K_{11}\xi \ln\frac{\xi}{\beta} \tag{S5.5}$$

A uniform director field indicated that ΔF must be negative for the meniscus tip within the characteristic length scale ξ. The cross-over from a uniform director field to a deformed director field at the characteristic length scale ξ suggests that the derivative of ΔF with respect to ξ must be zero:

$$\frac{\partial \Delta F}{\partial \xi} = 3W_{NI}\frac{\xi^2}{D_{\text{cap}}} - K_{11}\left(\ln\frac{\xi}{\beta} + 1\right) = 0 \tag{S5.6}$$

Since $\ln(\xi/\beta)$ is a weakly varying function of ξ, we ignored this term and obtained

$$\xi = \sqrt{\ln\frac{\xi}{\beta} + 1} \times \sqrt{\frac{K_{11}D_{\text{cap}}}{W_{NI}}} \sim \sqrt{\frac{K_{11}D_{\text{cap}}}{W_{NI}}} \tag{S5.7}$$

At first sight, this simplified scaling relationship is similar to the extrapolation length K_{11}/W_{NI} which indicates how strongly the surface anchoring affect the director field (*12*). We note however, that the characteristic length scale ξ has a different physical meaning by its definition, and it can be directly measured from POM images (fig. S23).

The effect of elasticity and surface anchoring on the characteristic length scale is straightforward: the value of ξ increases with increasing K_{11}, since a uniform director field reduces the elastic energy, and the value of ξ decreases with increasing W_{NI}, as the surface anchoring energy of the uniform director field is increased.

The scaling relationship also shows that the value of ξ is related to the diameter of the capillary, D_{cap}. By rescaling ξ with D_{cap}, we found the ratio

$$\frac{\xi}{D_{\text{cap}}} = \sqrt{\frac{K_{11}}{W_{NI}D_{\text{cap}}}} \tag{S5.8}$$

which is a dimensionless parameter that represents the relative characteristic length scale, that is, the relative size of the uniform director field in the meniscus tip. With decreasing D_{cap} (a stronger confinement effect), ξ decreases while ξ/D_{cap} increases. This relationship means that for narrower capillaries, the stronger confinement effect forces a larger relative size of the meniscus tip to adopt a uniform director field.

The dependence of the dimensionless parameter ξ/D_{cap} on D_{cap} is not unexpected, because the scaling analysis is based on the free energy argument that the competing elastic energy, F_E, and the surface anchoring energy, F_S, which scale with D_{cap} and D_{cap}^2, respectively. Then, the relative magnitude of the elastic energy to the surface anchoring energy, F_E/F_S, scales inversely with

D_{cap}. With decreasing D_{cap}, that is, a stronger confinement effect, the elastic energy dominates over the surface anchoring energy. Thus, in narrower capillaries, a larger relative volume of the nematic phase will adopt a uniform director field to reduce the elastic energy.

Experimentally, we measured ξ and calculated ξ/D_{cap} for the capillaries with D_{cap} varying from 100 to 400 µm (fig. S23). The diagrams in fig. S24 show that our experimental results qualitatively agree with scaling predictions, which are presented as dashed gray curves for eye guidance. We note however, that we cannot perform any quantitative fitting of the experimental results to extract the values of K_{11} or W_{NI}. The purpose of comparing ξ and ξ/D_{cap} of the capillaries with varying diameters is to verify whether the results collected in the experimentally accessible range of D_{cap} can show the predicted scaling behaviors, at least qualitatively.

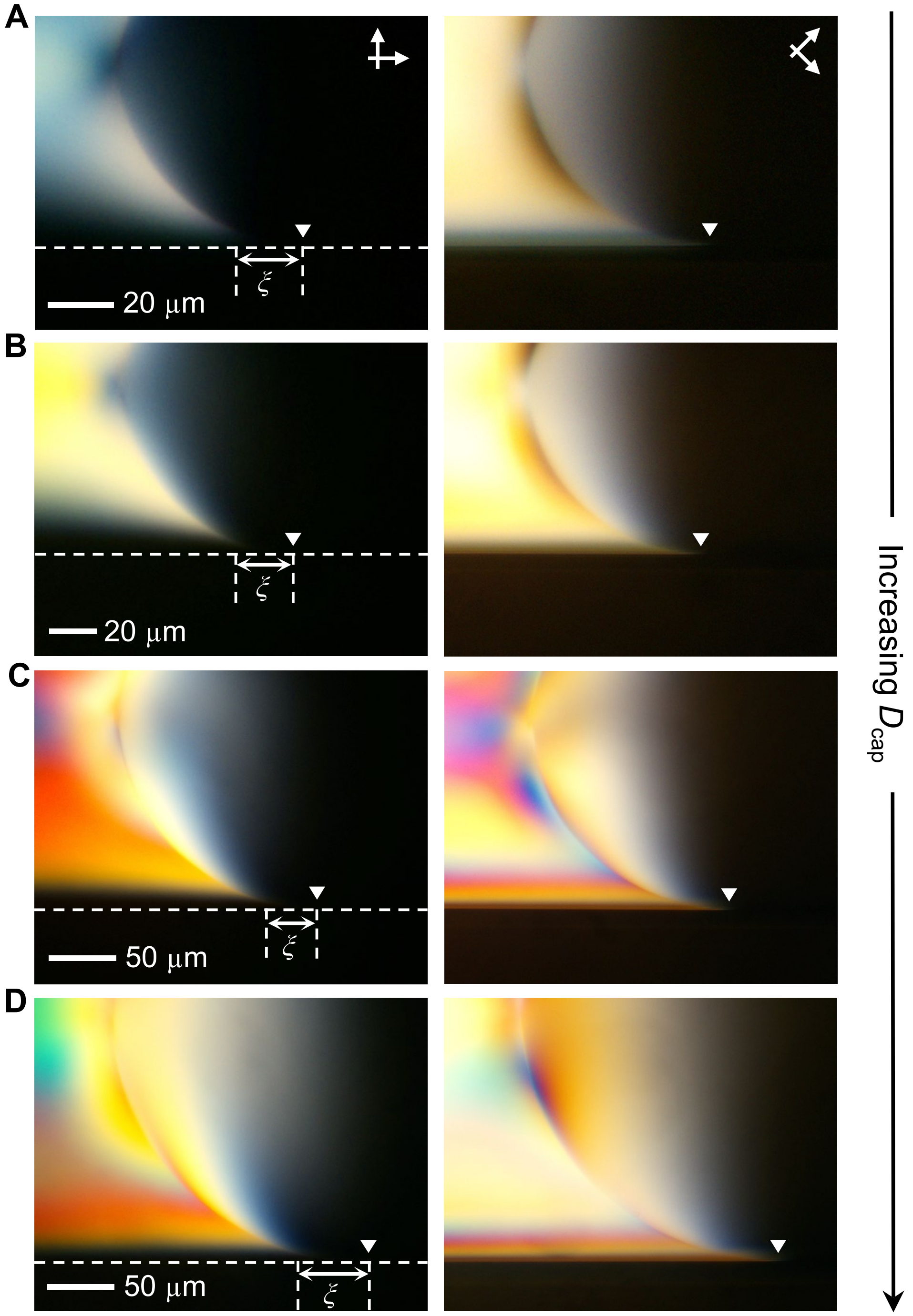

Fig. S23. POM images of the meniscus tip in capillaries with different diameters. (**A**) D_{cap} = 100 µm. (**B**) D_{cap} = 141 µm. (**C**) D_{cap} = 282 µm. (**D**) D_{cap} = 400 µm. The dashed white lines outline the glass surface. The location of the contact line is marked with white triangles. The capillaries were aligned parallel to the polarizer (left) and rotated by 45° (right). ξ is the characteristic length scale. C_P = 1.7 wt%.

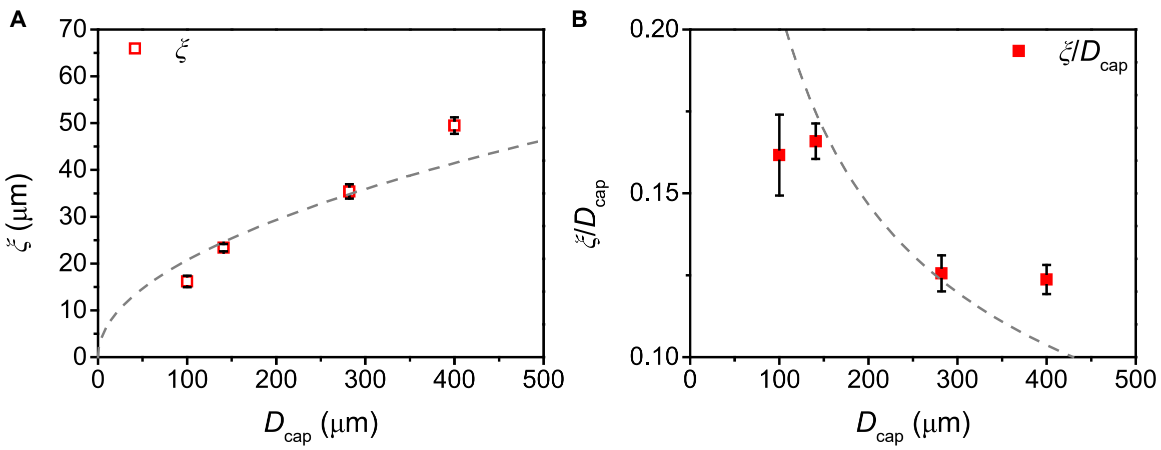

Fig. S24. Characteristic length scale ξ and the ratio ξ/D_{cap}. (**A**) Variation in the characteristic length scale ξ. (**B**) Variation in the ratio ξ/D_{cap}. Both (A) and (B) are plotted as functions of the capillary diameter D_{cap}. The dashed gray curves show the scaling predictions and are for eye guidance only. The error bars are the standard deviations of the results obtained from 5 meniscus tips.

Section S6. Interfacial tension of the nematic/solid interface

Considering a simple model of the nematic phase of long rigid rods of length L_{rod} confined to a cylindrical capillary of diameter D_{cap}. the excluded volume interactions between the rods and the solid surface create a depletion zone near the solid surface, whose width δ depends on the orientation of the rods due to the curvature of the solid surface (fig. S25). The scaling ansatz of the interfacial tension of the NS interface γ_{NS} related to the width of the depletion zone is written as (*57*):

$$\gamma_{NS} \simeq p\delta \tag{S6.1}$$

where p is the osmotic pressure of the nematic phase. We stress that this scaling relation is based on the thermodynamic argument that the increase in surface tension equals the work per unit area required to create a depletion zone of width δ against the osmotic pressure of the solution phase (*57*). For our experimental system involving PBDT solutions confined to glass capillaries, the scaling ansatz only accounts for the anisotropic excluded volume interactions between the PBDT rods and the curved glass surface. The elastic energy, surface anchoring energy, and specific interactions between the rods and the glass surface are not included.

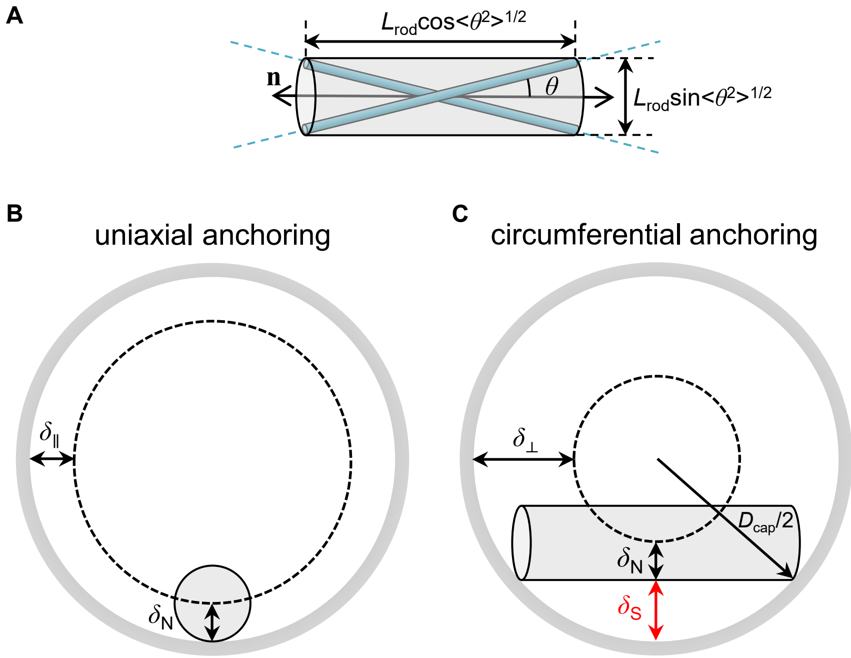

Fig. S25. Schematics of the depletion zone created by the excluded volume interactions between the rods and the curved solid surface. (**A**) Orientational fluctuation of the rigid rods. (**B**) Depletion zone $\delta_{\parallel}$ in the case of uniaxial anchoring. (**C**) Depletion zone of width $\delta_{\perp}$ in the case of circumferential anchoring.

Here we compare the situation in which the rods are uniaxially aligned parallel (∥) to the capillary long axis (fig. S25B), with that where the rods are aligned along the circumference of the capillary, that is, perpendicular (⊥) to the capillary long axis (fig. S25C). Due to the fluctuation of rod orientation, the rods sweep out a cone with diameter $L_{rod}\sin\langle\theta^2\rangle^{1/2}$, where $\langle\theta^2\rangle^{1/2}$ is the root-mean-square angle between the rods and the director (fig. S25A). In the highly

ordered nematic state, the orientation distribution of the rods is narrow, that is, $<\theta^2>^{1/2}$ is small. Thus, in the case of uniaxial anchoring, the width of the depletion zone $\delta_{\|}$ equals the distance δ_N from the center of the mass of the rods to the solid surface:

$$\delta_{\|} = \delta_N = \frac{1}{2} L_{rod} \sin\left\langle \theta^2 \right\rangle^{1/2} \simeq \frac{1}{2} L_{rod} \left\langle \theta^2 \right\rangle^{1/2} \quad \text{(S6.2)}$$

In the case of circumferential anchoring, the width of the depletion zone increases to $\delta_{\perp} = \delta_N + \delta_S$, where δ_S can be estimated from the geometry:

$$\delta_S = \frac{D_{cap}}{2} - \sqrt{\left(\frac{D_{cap}}{2}\right)^2 - \left(\frac{L_{rod} \cos\left\langle \theta^2 \right\rangle^{1/2}}{2}\right)^2} \simeq \frac{L_{rod}^2 \cos^2\left\langle \theta^2 \right\rangle^{1/2}}{4D_{cap}} \quad \text{(S6.3)}$$

Following the scaling ansatz, the difference in the interfacial tension is

$$\frac{\gamma_{NS}^{\perp} - \gamma_{NS}^{\|}}{\gamma_{NS}^{\|}} \simeq \frac{p\delta_{\perp} - p\delta_{\|}}{p\delta_{\|}} = \frac{p(\delta_N + \delta_S) - p\delta_N}{p\delta_N} = \frac{\delta_S}{\delta_N} \simeq \frac{L_{rod} \cos^2\left\langle \theta^2 \right\rangle^{1/2}}{2D_{cap}\left\langle \theta^2 \right\rangle^{1/2}} \quad \text{(S6.4)}$$

By applying Gaussian approximation to the orientation distribution of the rods under conditions of isotropic-nematic coexistence, $<\theta^2>^{1/2}$ was found to be approximately 0.245 rad (*11*). Thus, we obtain the difference in the interfacial tension as

$$\frac{\gamma_{NS}^{\perp} - \gamma_{NS}^{\|}}{\gamma_{NS}^{\|}} \simeq \frac{L_{rod} \cos^2\left\langle \theta^2 \right\rangle^{1/2}}{2D_{cap}\left\langle \theta^2 \right\rangle^{1/2}} \simeq \frac{3.8L_{rod}}{2D_{cap}} \simeq \frac{L_{rod}}{R_{cap}} \quad \text{(S6.5)}$$

where R_{cap} is the radius of curvature of the capillary. For PBDT rods, since L_{rod} (~100 nm) is small compared to R_{cap} (~50 μm), the difference in the interfacial energy is small. Nonetheless, the curvature of the surface indeed breaks the degeneracy of the planar surface anchoring, favoring rod alignment along the capillary long axis, which has the lowest curvature. This is different from chromonic liquid crystals where the large saddle-splay constant K_{24} favors rods alignment along the direction with the highest curvature, that is, the circumference of the capillary.

It should be pointed out that for this simple scaling analysis we assumed that the rods are rigid. This is true for PBDT rods since L_{rod} (~100 nm) is very much smaller than the persistence length (~1 μm). In comparison, the persistence lengths of Sunset Yellow (SSY) and disodium cromoglycate (DSCG), two commonly studied chromonic liquid crystals, are approximately 10 and 50 nm, respectively, and the average lengths of the aggregates formed by SSY and DSCG vary strongly with temperature and concentration, and are typically on the same order with their persistence lengths (*79, 80*). This suggests that chromonic liquid crystals are more flexible, as they are formed by dynamic non-covalent aggregation of small molecules. The flexibility of chromonic liquid crystals likely reduces the alignment force exerted by the surface curvature. Due to the structural differences in liquid crystal building blocks, our scaling analysis using a rigid rod model is more applicable to PBDT rods than to chromonic liquid crystals.

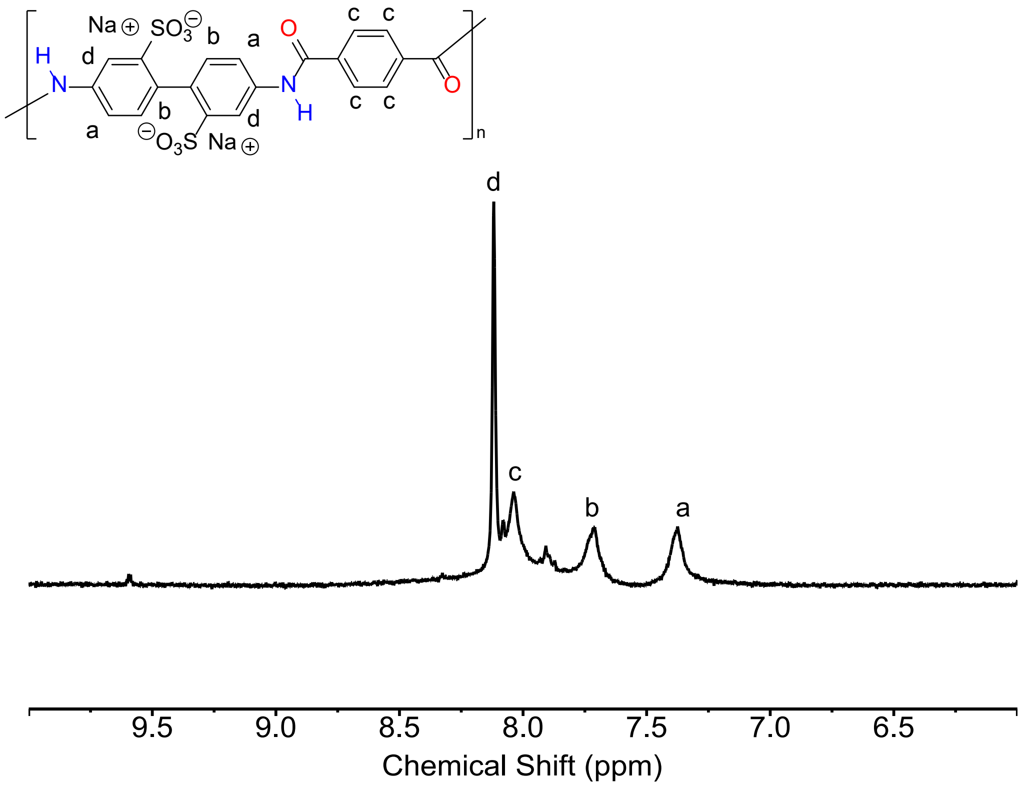

Fig. S26. ¹H-NMR spectrum of PBDT. The peaks in the range of 7.0-8.5 ppm were assigned to the aromatic protons (a-d) on the backbone of PBDT.

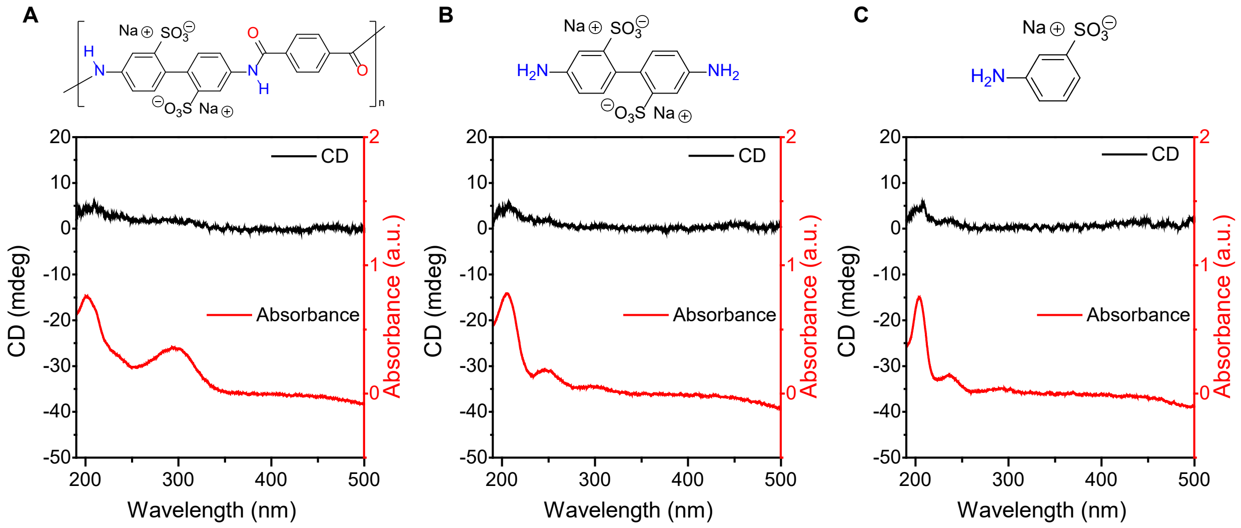

Fig. S27. CD and absorption spectra of PBDT, BDSA-Na, and a model compound. (**A**) The CD and absorption spectra of a 0.001 wt% aqueous solution of PBDT. (**B**) The CD and absorption spectra of a 0.001 wt% aqueous solution of BDSA-Na. (**C**) The CD and absorption spectra of a 0.001 wt% aqueous solution of sodium 3-aminobenzenesulfonate.

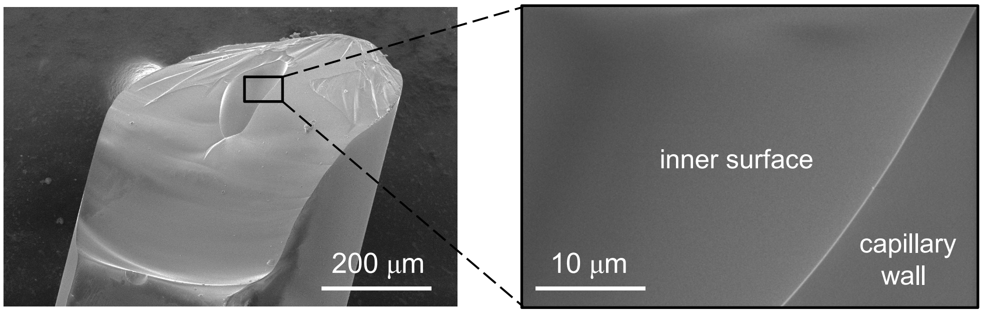

Fig. S28. Scanning electron microscopy (SEM) images of the inner surface of a 100 μm-diameter capillary. The enlarged image on the right shows a smooth inner surface of the glass capillary.

Movie S1. Formation of nematic segments at D_{cap} = 100 μm and C_P = 1.6 wt%. The movie recorded using POM shows the formation of nematic segments through the wetting and dewetting of the nematic film. The capillary was aligned 45° to the crossed polarizer and analyzer.

REFERENCES AND NOTES

1. D. Nepal, S. Kang, K. M. Adstedt, K. Kanhaiya, M. R. Bockstaller, L. C. Brinson, M. J. Buehler, P. V. Coveney, K. Dayal, J. A. El-Awady, L. C. Henderson, D. L. Kaplan, S. Keten, N. A. Kotov, G. C. Schatz, S. Vignolini, F. Vollrath, Y. Wang, B. I. Yakobson, V. V. Tsukruk, H. Heinz, Hierarchically structured bioinspired nanocomposites. *Nat. Mater.* **22**, 18–35 (2023).

2. Z. Wang, C. L. C. Chan, T. H. Zhao, R. M. Parker, S. Vignolini, Recent advances in block copolymer self-assembly for the fabrication of photonic films and pigments. *Adv. Opt. Mater.* **9**, 2100519 (2021).

3. A. Shi, B. Li, Self-assembly of diblock copolymers under confinement. *Soft Matter* **9**, 1398–1413 (2013).

4. R. M. Michell, A. J. Müller, Confined crystallization of polymeric materials. *Prog. Polym. Sci.* **54-55**, 183–213 (2016).

5. M. Li, S. Xu, E. Kumacheva, Convection in polymeric fluids subjected to vertical temperature gradients. *Macromolecules* **33**, 4972–4978 (2000).

6. S. Xu, E. Kumacheva, Ordered morphologies in polymeric films produced by replication of convection patterns. *J. Am. Chem. Soc.* **124**, 1142–1143 (2002).

7. V. Cherpak, V. F. Korolovych, R. Geryak, T. Turiv, D. Nepal, J. Kelly, T. J. Bunning, O. D. Lavrentovich, W. T. Heller, V. V. Tsukruk, Robust chiral organization of cellulose nanocrystals in capillary confinement. *Nano Lett.* **18**, 6770–6777 (2018).

8. A. Nikoubashman, Ordering, phase behavior, and correlations of semiflexible polymers in confinement. *J. Chem. Phys.* **154**, 090901 (2021).

9. M. M. C. Tortora, D. Jost, Orientational wetting and topological transitions in confined solutions of semiflexible polymers. *Macromolecules* **56**, 1339–1351 (2023).

10. L. Onsager, The effects of shape on the interaction of colloidal particles. *Ann. N. Y. Acad. Sci.* **51**, 627–659 (1949).

11. T. Odijk, Theory of lyotropic polymer liquid crystals. *Macromolecules* **19**, 2313–2329 (1986).

12. P.-G. de Gennes, J. Prost, *The Physics of Liquid Crystals* (Oxford Univ. Press Inc., 1993).

13. M. M. C. Tortora, G. Mishra, D. Prešern, J. P. K. Doye, Chiral shape fluctuations and the origin of chirality in cholesteric phases of DNA origamis. *Sci. Adv.* **6**, eaaw8331 (2020).

14. G. Nyström, M. Arcari, R. Mezzenga, Confinement-induced liquid crystalline transitions in amyloid fibril cholesteric tactoids. *Nat. Nanotechnol.* **13**, 330–336 (2018).

15. Y. Li, J. J.-Y. Suen, E. Prince, E. M. Larin, A. Klinkova, H. Thérien-Aubin, S. Zhu, B. Yang, A. S. Helmy, O. D. Lavrentovich, Colloidal cholesteric liquid crystal in spherical confinement. *Nat. Commun.* **7**, 12520 (2016).

16. Y. Li, E. Prince, S. Cho, A. Salari, Y. M. Golestani, O. D. Lavrentovich, E. Kumacheva, Periodic assembly of nanoparticle arrays in disclinations of cholesteric liquid crystals. *Proc. Natl. Acad. Sci. U.S.A.* **114**, 2137–2142 (2017).

17. Y. Li, N. Khuu, E. Prince, M. Alizadehgiashi, E. Galati, O. D. Lavrentovich, E. Kumacheva, Nanoparticle-laden droplets of liquid crystals: Interactive morphogenesis and dynamic assembly. *Sci. Adv.* **5**, eaav1035 (2019).

18. E. Prince, Y. Wang, I. I. Smalyukh, E. Kumacheva, Cylindrical confinement of nanocolloidal cholesteric liquid crystal. *J. Phys. Chem. B* **125**, 8243–8250 (2021).

19. T. G. Parton, R. M. Parker, G. T. van de Kerkhof, A. Narkevicius, J. S. Haataja, B. Frka-Petesic, S. Vignolini, Chiral self-assembly of cellulose nanocrystals is driven by crystallite bundles. *Nat. Commun.* **13**, 2657 (2022).

20. G. Fittolani, D. Vargová, P. H. Seeberger, Y. Ogawa, M. Delbianco, Bottom-up approach to understand chirality transfer across scales in cellulose assemblies. *J. Am. Chem. Soc.* **144**, 12469–12475 (2022).

21. E. Grelet, M. M. C. Tortora, Elucidating chirality transfer in liquid crystals of viruses. *Nat. Mater.* **23**, 1276–1282 (2024).

22. R. Chang, "Chiral configurations from achiral lyotropic chromonic liquid crystals under confinements," thesis, Georgia Institute of Technology, Atlanta, GA (2018).

23. J. Lv, D. Ding, X. Yang, K. Hou, X. Miao, D. Wang, B. Kou, L. Huang, Z. Tang, Biomimetic chiral photonic crystals. *Angew. Chem. Int. Ed. Engl.* **58**, 7783–7787 (2019).

24. K. S. Park, Z. Xue, B. B. Patel, H. An, J. J. Kwok, P. Kafle, Q. Chen, D. Shukla, Y. Diao, Chiral emergence in multistep hierarchical assembly of achiral conjugated polymers. *Nat. Commun.* **13**, 2738 (2022).

25. Z. Yang, Y. Wei, J. Wei, Z. Yang, Chiral superstructures of inorganic nanorods by macroscopic mechanical grinding. *Nat. Commun.* **13**, 5844 (2022).

26. D. Revignas, A. Ferrarini, Spontaneous twisting of achiral hard rod nematics. *Phys. Rev. Lett.* **130**, 028102 (2023).

27. A. V. Gonzalez, M. Gonzalez, T. Hanrath, Emergence and inversion of chirality in hierarchical assemblies of CdS nanocrystal fibers. *Sci. Adv.* **9**, eadi5520 (2023).

28. T. J. Ugras, R. B. Carson, R. P. Lynch, H. Li, Y. Yao, L. Cupellini, K. A. Page, D. Wang, A. Arbe, S. Bals, L. Smieska, A. R. Woll, O. Arteaga, T. Jávorfi, G. Siligardi, G. Pescitelli, S. J. Weinstein, R. D. Robinson, Transforming achiral semiconductors into chiral domains with exceptional circular dichroism. *Science* **387**, eado7201 (2025).

29. C. F. Dietrich, P. Rudquist, K. Lorenz, F. Giesselmann, Chiral structures from achiral micellar lyotropic liquid crystals under capillary confinement. *Langmuir* **33**, 5852–5862 (2017).

30. L. Tortora, O. D. Lavrentovich, Chiral symmetry breaking by spatial confinement in tactoidal droplets of lyotropic chromonic liquid crystals. *Proc. Natl. Acad. Sci. U.S.A.* **108**, 5163–5168 (2011).

31. A. Nych, U. Ognysta, I. Muševič, D. Seč, M. Ravnik, S. Žumer, Chiral bipolar colloids from nonchiral chromonic liquid crystals. *Phys. Rev. E* **89**, 062502 (2014).

32. J. Jeong, Z. S. Davidson, P. J. Collings, T. C. Lubensky, A. G. Yodh, Chiral symmetry breaking and surface faceting in chromonic liquid crystal droplets with giant elastic anisotropy. *Proc. Natl. Acad. Sci. U.S.A.* **111**, 1742–1747 (2014).

33. J. Jeong, L. Kang, Z. S. Davidson, P. J. Collings, T. C. Lubensky, A. G. Yodh, Chiral structures from achiral liquid crystals in cylindrical capillaries. *Proc. Natl. Acad. Sci. U.S.A.* **112**, E1837–E1844 (2015).

34. Z. S. Davidson, L. Kang, J. Jeong, T. Still, P. J. Collings, T. C. Lubensky, A. G. Yodh, Chiral structures and defects of lyotropic chromonic liquid crystals induced by saddle-splay elasticity. *Phys. Rev. E* **91**, 050501 (2015).

35. K. Nayani, R. Chang, J. Fu, P. W. Ellis, A. Fernandez-Nieves, J. O. Park, M. Srinivasarao, Spontaneous emergence of chirality in achiral lyotropic chromonic liquid crystals confined to cylinders. *Nat. Commun.* **6**, 8067 (2015).

36. G. Park, S. Čopar, A. Suh, M. Yang, U. Tkalec, D. K. Yoon, Periodic arrays of chiral domains generated from the self-assembly of micropatterned achiral lyotropic chromonic liquid crystal. *ACS Cent. Sci.* **6**, 1964–1970 (2020).

37. Q. Zhang, W. Wang, S. Zhou, R. Zhang, I. Bischofberger, Flow-induced periodic chiral structures in an achiral nematic liquid crystal. *Nat. Commun.* **15**, 7 (2024).

38. S. Varytimiadou, D. Revignas, F. Giesselmann, A. Ferrarini, Elasticity of lyotropic nematic liquid crystals: A review of experiments, theory and simulation. *Liq. Cryst. Rev.* **12**, 57–104 (2024).

39. R. B. Meyer, “Macroscopic phenomena in nematic polymers” in *Polymer Liquid Crystals*, A. Ciferri, W. R. Krigbaum, R. B. Meyer, Eds. (Academic Press, Inc., 1982), pp. 133–163.

40. R. B. Meyer, F. Lonberg, V. Taratuta, S. Fraden, S.-D. Lee, A. J. Hurd, Measurements of the anisotropic viscous and elastic properties of lyotropic polymer nematics. *Faraday Discuss. Chem. Soc.* **79**, 125–132 (1985).

41. T. Drwenski, S. Dussi, M. Hermes, M. Dijkstra, R. van Roij, Phase diagrams of charged colloidal rods: Can a uniaxial charge distribution break chiral symmetry? *J. Chem. Phys.* **144**, 094901 (2016).

42. R. D. Williams, Two transitions in tangentially anchored nematic droplets. *J. Phys. A Math. Gen.* **19**, 3211–3222 (1986).

43. P. Prinsen, P. van der Schoot, Parity breaking in nematic tactoids. *J. Phys. Condens. Matter* **16**, 8835–8850 (2004).

44. N. Sarkar, L. D. Kershner, Rigid rod water-soluble polymers. *J. Appl. Polym. Sci.* **62**, 393–408 (1996).

45. Z. L. Wu, M. Arifuzzaman, T. Kurokawa, K. Le, J. Hu, T. L. Sun, H. Furukawa, H. Masunaga, J. P. Gong, Supramolecular assemblies of a semirigid polyanion in aqueous solutions. *Macromolecules* **46**, 3581–3586 (2013).

46. Y. Wang, Y. He, Z. Yu, J. Gao, S. ten Brinck, C. Slebodnick, G. B. Fahs, C. J. Zanelotti, M. Hegde, R. B. Moore, B. Ensing, T. J. Dingemans, R. Qiao, L. A. Madsen, Double helical conformation and extreme rigidity in a rodlike polyelectrolyte. *Nat. Commun.* **10**, 801 (2019).

47. Y. Bouligand, J. P. Denefle, J. P. Lechaire, M. Maillard, Twisted architectures in cell-free assembled collagen gels: Study of collagen substrates used for cultures. *Biol. Cell* **54**, 143–162 (1985).

48. S. Ehrig, B. Schamberger, C. M. Bidan, A. West, C. Jacobi, K. Lam, P. Kollmannsberger, A. Petersen, P. Tomancak, K. Kommareddy, F. D. Fischer, P. Fratzl, J. W. C. Dunlop, Surface tension determines tissue shape and growth kinetics. *Sci. Adv.* **5**, eaav9394 (2019).

49. F. Livolant, A. Leforestier, Chiral discotic columnar germs of nucleosome core particles. *Biophys. J.* **78**, 2716–2729 (2000).

50. N. Chaturvedi, R. D. Kamien, Gnomonious projections for bend-free textures: Thoughts on the splay-twist phase. *Proc. Math. Phys. Eng. Sci.* **476**, 20190824 (2020).

51. J. M. Miller, D. Hall, J. Robaszewski, P. Sharma, M. F. Hagan, G. M. Grason, Z. Dogic, All twist and no bend makes raft edges splay: Spontaneous curvature of domain edges in colloidal membranes. *Sci. Adv.* **6**, eaba2331 (2020).

52. A. V. Dobrynin, M. Rubinstein, Theory of polyelectrolytes in solutions and at surfaces. *Prog. Polym. Sci.* **30**, 1049–1118 (2005).

53. H. H. Wensink, G. J. Vroege, Isotropic-nematic phase behavior of length-polydisperse hard rods. *J. Chem. Phys.* **119**, 6868–6882 (2003).

54. A. J. Liu, D. J. Durian, E. Herbolzheimer, S. A. Safran, Wetting transitions in a cylindrical pore. *Phys. Rev. Lett.* **65**, 1897–1900 (1990).

55. H. Tanaka, Dynamic interplay between phase separation and wetting in a binary mixture confined in a one-dimensional capillary. *Phys. Rev. Lett.* **70**, 53–56 (1993).

56. R. H. Tromp, S. Lindhoud, Arrested segregative phase separation in capillary tubes. *Phys. Rev. E* **74**, 031604 (2006).

57. P. van der Schoot, Remarks on the interfacial tension in colloidal systems. *J. Phys. Chem. B* **103**, 8804–8808 (1999).

58. V. Jamali, E. G. Biggers, P. van der Schoot, M. Pasquali, Line tension of twist-free carbon nanotube lyotropic liquid crystal microdroplets on solid surfaces. *Langmuir* **33**, 9115–9121 (2017).

59. V. Jamali, N. Behabtu, B. Senyuk, J. A. Lee, I. I. Smalyukh, P. van der Schoot, M. Pasquali, Experimental realization of crossover in shape and director field of nematic tactoids. *Phys. Rev. E* **91**, 042507 (2015).

60. M. Dijkstra, R. van Roij, R. Evans, Wetting and capillary nematization of a hard-rod fluid: A simulation study. *Phys. Rev. E* **63**, 051703 (2001).

61. D. van der Beek, H. Reich, P. van der Schoot, M. Dijkstra, T. Schilling, R. Vink, M. Schmidt, R. van Roij, H. Lekkerkerker, Isotropic-nematic interface and wetting in suspensions of colloidal platelets. *Phys. Rev. Lett.* **97**, 087801 (2006).

62. R. H. J. Otten, P. van der Schoot, Capillary rise of an isotropic-nematic fluid interface: Surface tension and anchoring versus elasticity. *Langmuir* **25**, 2427–2436 (2009).

63. J. R. L. Cousins, A. S. Bhadwal, L. T. Corson, B. R. Duffy, I. C. Sage, C. V. Brown, N. J. Mottram, S. K. Wilson, Weak-anchoring effects in a thin pinned ridge of nematic liquid crystal. *Phys. Rev. E* **107**, 034702 (2023).

64. A. D. Rey, Capillary models for liquid crystal fibers, membranes, films, and drops. *Soft Matter* **3**, 1349–1368 (2007).

65. S. Wolfsheimer, C. Tanase, K. Shundyak, R. van Roij, T. Schilling, Isotropic-nematic interface in suspensions of hard rods: Mean-field properties and capillary waves. *Phys. Rev. E* **73**, 061703 (2006).

66. W.-J. Chung, J.-W. Oh, K. Kwak, B. Y. Lee, J. Meyer, E. Wang, A. Hexemer, S.-W. Lee, Biomimetic self-templating supramolecular structures. *Nature* **478**, 364–368 (2011).

67. J. V. Selinger, Interpretation of saddle-splay and the Oseen-Frank free energy in liquid crystals. *Liq. Cryst. Rev.* **6**, 129–142 (2018).

68. G. Napoli, L. Vergori, Extrinsic curvature effects on nematic shells. *Phys. Rev. Lett.* **108**, 207803 (2012).

69. H. Fu, J. Huang, J. J. B. van der Tol, L. Su, Y. Wang, S. Dey, P. Zijlstra, G. Fytas, G. Vantomme, P. Y. W. Dankers, E. W. Meijer, Supramolecular polymers form tactoids through liquid–liquid phase separation. *Nature* **626**, 1011–1018 (2024).

70. E. Paineau, M.-E. M. Krapf, M.-S. Amara, N. V. Matskova, I. Dozov, S. Rouzière, A. Thill, P. Launois, P. Davidson, A liquid-crystalline hexagonal columnar phase in highly-dilute suspensions of imogolite nanotubes. *Nat. Commun.* **7**, 10271 (2016).

71. J. Kim, S. Michelin, M. Hilbers, L. Martinelli, E. Chaudan, G. Amselem, E. Fradet, J.-P. Boilot, A. M. Brouwer, C. N. Baroud, J. Peretti, T. Gacoin, Monitoring the orientation of rare-earth-doped nanorods for flow shear tomography. *Nat. Nanotechnol.* **12**, 914–919 (2017).

72. O. Oki, C. Kulkarni, H. Yamagishi, S. C. J. Meskers, Z.-H. Lin, J.-S. Huang, E. W. Meijer, Y. Yamamoto, Robust angular anisotropy of circularly polarized luminescence from a single twisted-bipolar polymeric microsphere. *J. Am. Chem. Soc.* **143**, 8772–8779 (2021).

73. Q. Liu, Y. Cui, D. Gardner, X. Li, S. He, I. I. Smalyukh, Self-alignment of plasmonic gold nanorods in reconfigurable anisotropic fluids for tunable bulk metamaterial applications. *Nano Lett.* **10**, 1347–1353 (2010).

74. J. Lv, K. Hou, D. Ding, D. Wang, B. Han, X. Gao, M. Zhao, L. Shi, J. Guo, Y. Zheng, X. Zhang, C. Lu, L. Huang, W. Huang, Z. Tang, Gold nanowire chiral ultrathin films with ultrastrong and broadband optical activity. *Angew. Chem. Int. Ed. Engl.* **56**, 5055–5060 (2017).

75. J. Lv, X. Yang, Z. Tang, Rational design of all-inorganic assemblies with bright circularly polarized luminescence. *Adv. Mater.* **35**, e2209539 (2023).

76. M. Ravnik, S. Žumer, Landau-de Gennes modelling of nematic liquid crystal colloids. *Liq. Cryst.* **36**, 1201–1214 (2009).

77. J.-B. Fournier, P. Galatola, Modeling planar degenerate wetting and anchoring in nematic liquid crystals. *Europhys. Lett.* **72**, 403–409 (2005).

78. S. Kumari, S. Dwivedi, R. Podgornik, On the nature of screening in Voorn–Overbeek type theories. *J. Chem. Phys.* **156**, 244901 (2022).

79. S. Zhou, Y. A. Nastishin, M. M. Omelchenko, L. Tortora, V. G. Nazarenko, O. P. Boiko, T. Ostapenko, T. Hu, C. C. Almasan, S. N. Sprunt, J. T. Gleeson, O. D. Lavrentovich, Elasticity of lyotropic chromonic liquid crystals probed by director reorientation in a magnetic field. *Phys. Rev. Lett.* **109**, 037801 (2012).

80. S. Zhou, K. Neupane, Y. A. Nastishin, A. R. Baldwin, S. V. Shiyanovskii, O. D. Lavrentovich, S. Sprunt, Elasticity, viscosity, and orientational fluctuations of a lyotropic chromonic nematic liquid crystal disodium cromoglycate. *Soft Matter* **10**, 6571–6581 (2014).
